# Effect of age and the individual on the gastrointestinal bacteriome of ponies fed a high-starch diet

**DOI:** 10.1371/journal.pone.0232689

**Published:** 2020-05-08

**Authors:** Philippa K. Morrison, Charles J. Newbold, Eleanor Jones, Hilary J. Worgan, Dai H. Grove-White, Alexandra H. Dugdale, Clare Barfoot, Patricia A. Harris, Caroline McG. Argo

**Affiliations:** 1 Scotland’s Rural College, Aberdeen, Scotland, United Kingdom; 2 Scotland’s Rural College, Edinburgh, Scotland, United Kingdom; 3 Institute of Biological, Environmental and Rural Sciences, Aberystwyth University, Aberystwyth, Wales, United Kingdom; 4 Faculty of Health and Life Sciences, University of Liverpool, Neston, Wirral, England, United Kingdom; 5 ChesterGates Veterinary Specialists CVS (UK) Ltd., Chester, England, United Kingdom; 6 MARS Horsecare UK Ltd, Buckinghamshire, England, United Kingdom; 7 WALTHAM Petcare Science Institute, Waltham-on-the-Wolds, Leicestershire, England, United Kingdom; Universidad Nacional Autonoma de Mexico Instituto de Investigaciones en Ecosistemas y Sustentabilidad, MEXICO

## Abstract

Bacteria residing in the gastrointestinal tract of mammals are crucial for the digestion of dietary nutrients. Bacterial community composition is modified by age and diet in other species. Although horses are adapted to consuming fibre-based diets, high-energy, often high-starch containing feeds are increasingly used. The current study assessed the impact of age on the faecal bacteriome of ponies transitioning from a hay-based diet to a high-starch diet. Over two years, 23 Welsh Section A pony mares were evaluated (Controls, 5–15 years, n = 6/year, 12 in total; Aged, ≥19 years, n = 6 Year 1; n = 5 Year 2, 11 in total). Across the same 30-week (May to November) period in each year, animals were randomly assigned to a 5-week period of study and were individually fed the same hay to maintenance (2% body mass as daily dry matter intake) for 4-weeks. During the final week, 2g starch per kg body mass (micronized steam-flaked barley) was incorporated into the diet (3-day transition and 5 days at maximum). Faecal samples were collected for 11 days (final 3 days hay and 8 days hay + barley feeding). Bacterial communities were determined using Ion Torrent Sequencing of amplified V1–V2 hypervariable regions of 16S rRNA. Age had a minimal effect on the bacteriome response to diet. The dietary transition increased *Candidatus Saccharibacteria* and *Firmicutes* phyla abundance and reduced *Fibrobactres* abundance. At the genera level, *Streptococcus* abundance was increased but not consistently across individual animals. Bacterial diversity was reduced during dietary transition in *Streptococcus* ‘responders’. Faecal pH and VFA concentrations were modified by diet but considerable inter-individual variation was present. The current study describes compositional changes in the faecal bacteriome associated with the transition from a fibre-based to a high-starch diet in ponies and emphasises the individual nature of dietary responses, which may reflect functional differences in the bacterial populations present in the hindgut.

## Introduction

For all mammals, the symbiotic relationship that exists between the host and the vast community of microorganisms inhabiting the gastrointestinal tract is vital in maintaining whole-body homeostasis, through the provision and digestion of dietary nutrients. Horses and ponies have evolved to consume a largely fibre-based diet [1,2] and rely upon the microbial fermentation of dietary fibre in the large intestine to provide energy in the form of short-chain fatty acids including acetate, butyrate and propionate. The equine gastrointestinal microbiome has been well characterised, and like humans, appears to be dominated by bacteria belonging to the *Firmicutes* and *Bacteroidetes* phyla [3–5].

As for other species, the community structure of the gastrointestinal microbiome in horses can be significantly modified by diet, with the most marked changes appearing to follow the introduction of starch to a fibre-based diet [6,7]. Significant changes in the gastrointestinal bacterial population of horses and ponies have been clearly demonstrated following the introduction of a high-starch diet using both culture-dependent and–independent techniques [8,9]. The incorporation of starch in the diet of horses and ponies that exceeds the capacity for digestion in the small intestine can result in undigested starch reaching the hindgut. As a consequence, the fermentation of dietary starch in the hindgut of horses by resident bacterial populations has been shown to lead to increases in the abundance of amylolytic bacteria including *Lactobacilli* and *Streptococcus* and reductions in cellulolytic bacterial populations [8,10]. These changes in the bacterial populations are associated with a reduction in gastrointestinal pH due to increases in lactate production, and may lead to the development of conditions such as colic and laminitis in susceptible animals. However, the microbial response to starch feeding is not consistent between animals, with the extent of the microbial response appearing to vary between individual animals [11].

Furthermore, both the botanical source and feed processing of starch have been shown to impact upon starch digestion [12,13]. Feeding thermo-mechanical forms of barley as opposed to whole grain or mechanically-treated forms of barley has been shown to result in greater ADF (acid detergent fibre) digestibility, and suggests that feeding thermo-mechanical forms of barley may limit the amount of undigested starch reaching the hindgut [14,15].

In addition to diet, age also impacts on the composition of the gastrointestinal microbiome and has previously been associated with a reduction in bacterial diversity in human studies [16]. However this has not always been a consistent finding in equine studies [4,9]. Improvements in husbandry are considered to have contributed to an increasing population of aged horses and ponies within the UK leisure sector [17]. Although current, UK-wide data is lacking, the most recent demographic study identified that aged animals (≥ 15 years) accounted for 29% of the study population of leisure horses and ponies residing in Northern England and North Wales [18]. With increases in the aged population of horses and ponies comes a requirement to further our understanding of nutritional requirements and responses in these animals. Whilst fibre remains the mainstay of nutritional requirements, cereals may be incorporated into diets for those animals requiring additional energy to maintain bodyweight. Therefore, the current study was designed to evaluate the impact of age on the response of the faecal bacteriome to the incorporation of starch (barley) into a forage-based (hay) diet. It was hypothesised that following the incorporation of starch into the diet, aged ponies would demonstrate a more marked perturbation of the faecal microflora with greater reductions in bacterial diversity compared to control animals.

## Materials and methods

### Animals and husbandry

Twenty-three Welsh Section A pony mares were studied at the same facility over the same 30-week period (May-December) across two consecutive years (Year 1 [2015] n = 12; Year 2 [2016] n = 11). The animals were privately-owned and recruited within a 30-mile radius of the University of Liverpool’s Ness Heath Farm. Recruited animals met selection criteria (below) and were clinically evaluated to be in good general and oral health. Animals had not received any antibiotic treatment in the previous 60 days prior to the commencement of or at any time during the study. Basal adrenocorticotrophic hormone (ACTH) concentrations were measured as a biomarker for pituitary *pars intermedia* dysfunction in plasma samples (Siemens Immulite 1000R, Chemiluminescent Assay) obtained prior to the commencement of the study. Data for all animals were within the normal seasonal range [19]. All animals were given appropriate anthelmintic treatment prior to the commencement of the study and routine foot care, vaccination and anthelmintic protocols were maintained throughout the study. In recognition of the potential impact anthelmintic treatment might have on the gut microbiome composition, all anthelmintic treatment was provided during the first of four weeks on the ‘hay washout’, therefore it was expected that any initial changes in bacterial composition or diversity caused by the treatment would be reversed before faecal samples were collected at the end of the hay washout period [20,21].

Animals were recruited into one of two groups based on age (Control animals (*n* = 6/year) were aged between 5–15 years with a body condition score (BCS) between 4.5-6/9 [22] and aged animals (n = 6 Year 1; n = 5 Year 2) were ≥19 years old (Table 1).

**Table 1:** Outset phenotype data (mean ± SD) for animals in the current study.

Group	Number of animals	Age	BM (kg)	BCS (/9)	% Body Fat	Height (cm)
**Control**	12	9.83 ± 3.21	237.50 ± 23.21	4.99 ± 0.83	9.59 ± 6.65	117.53 ± 3.67
**Aged**	11	21.54 ± 2.94	253.23 ± 27.43	5.95 ± 1.30	15.69 ± 7.25	117.64 ± 2.62

Outset data (mean ± SD) are presented for animal age (years), body mass (BM, kg), withers height (cm), body condition score (BCS, 9-point scale) and body fat percentage (as calculated following deuterium oxide dilution).

All procedures were conducted in accordance with Home Office (ASPA) requirements and approved by the University of Liverpool’s Animal Welfare Committee (Home Office project license number: PPL 70/8475). Written informed consent was obtained from all owners.

### Study design

In each year, the 30-week study was divided into six consecutive 5 week study-periods. Each year, animals were randomly assigned to a single study-period, during which time they were ‘on-study’ and were individually housed in loose boxes (3m x 5m), bedded on wood shavings and had free access to fresh water at all times. Where possible, animals were allowed pasture access for 30 minutes daily to permit basal exercise and social contact. Grazing was inhibited by muzzles (Comfort Grazing Muzzle, Shires UK). When ‘off study’ animals were kept at pasture. When ‘on-study’ during the 5-week study-period, animals were fed grass hay from the same batch for 4 weeks (27 days; ‘hay washout’) at a level designed to approximate maintenance (2% body mass (BM) as dry matter (DM) daily). Additionally, a nutrient balancer (SPILLERS^™^ Lite and Lean Balancer, UK) was fed daily to 0.1% BM. In recognition of routine husbandry practices, hay allowances for each animal were equally divided and offered as two daily meals (08.30h and 16.30h). The nutrient balancer was damped and fed once daily (08.30h). Individual feed requirement was set according to entry BM to the study-period. During the final 8 days of the study-period, micronized steam-flaked barley (Masham Micronised Feeds) was introduced gradually over 3 days (25%, 50% and 75% of maximum individual requirement), followed by 5 days at maximum individual requirement. Maximum individual requirements were calculated to provide 2g starch/kg BM daily as one meal (08.30h), fed in addition to daily hay and nutrient balancer provisions. The amount of starch fed during the final 5 days was chosen to induce a significant change in the gastrointestinal microflora and has been suggested to be the maximum amount of starch that can be fed safely in one meal [13,15]. Three replicate samples of each feedstuff were collected and independently analysed in triplicate. Mean compositions for key nutrients can be found in S1 Table.

### Physical measurements

The BCS, measures of heart and belly girth and BM of all ponies were recorded weekly (BM, nearest 500g, Lightweight Intermediate; Horseweigh, UK–which had been regularly calibrated; BCS [22]). BCS and circumferential measures were performed by the same observer on each occasion.

### Faecal collection

Fresh faecal samples were collected daily for eleven consecutive days from each individual animal. Samples were collected during the final 3 days of hay feeding (days 25–27, inclusive), whilst the remaining 8 samples were collected during the three-day introduction to barley (days 28–30 inclusive) and the five-days of maximum barley provision (days 31–35 inclusive).

The first defecation spontaneously voided by each animal (after 09.00) was immediately sampled from the centre portion with a gloved hand into a clean steel bowl to minimise environmental contamination. On collection, samples were hand-mixed and aliquoted into four, 5 ml sterile vials (Scientific Laboratory Supplies, UK). Vials were snap-frozen in liquid nitrogen within 5 minutes of collection and stored at -80°C prior to DNA extraction.

### Estimates of total body composition

Total body water (TBW) and total body fat mass were calculated for each individual animal during the final week of the hay feeding period using the deuterium oxide (D2O) dilution method, as previously described and validated for clinical use in the pony [23]. Deuterium enrichments in plasma samples were analysed in duplicate by a commercial laboratory (Iso-analytical, Cheshire, UK) by gas isotope ratio mass spectrometry.

### Combined Glucose-Insulin Tolerance Test (CGIT)

To generate individual animal indices of insulin sensitivity, a dynamic CGIT was performed on all animals during the final week of the hay feeding period [24]. Blood samples were immediately transferred to lithium heparinised tubes (BD vacutainer), mixed and placed on ice prior to centrifugation (2000g for 10 minutes). Plasma was aliquoted in duplicate and stored at -20°C prior to analysis. Plasma glucose samples were analysed using the hexokinase method. Plasma insulin concentrations were measured using a chemiluminescent assay (Siemens Immulite 1000R, Chemiluminescent Assay).

### Apparent digestibility

Total faecal collections were conducted for individual ponies over 3 consecutive days (72 hours) during the final week of the hay feeding period and repeated during the final 3 days of barley feeding. Any refused feed was recorded at the end of each 24-hour period. Total daily faecal collections were weighed, thoroughly mixed and duplicate samples were collected for analysis. Dry matter (DM) contents of faeces were determined by oven drying (70°C) duplicate samples (~250g) to constant mass. Ash contents were recorded following combustion of duplicate DM samples at 550°C (Carbolite OAF:1; Carbolite Furnaces). The three dry faecal samples collected over a 72-hour period were ground, pooled and homogenized for the evaluation of GE (gross energy) content (MJ/kgDM) by bomb calorimetry at a commercial laboratory (Sciantec, UK).

### DNA extraction

Genomic DNA was extracted from freeze-dried faecal samples (25 mg DM) which were bead-beaten in 4% SDS lysis buffer for 45 seconds. DNA was extracted using a CTAB/Chloroform method (adapted from [25]) in that lysis of cells was achieved by incubating with sodium dodecyl sulphate (SDS) buffer for 10 min at 95°C and potassium acetate was substituted for phenol in removing proteins. Concentrations and qualities of genomic DNA were assessed by spectrophotometry (Nanodrop ND-100, Thermo Scientific, USA).

### Ion torrent next generation sequencing

Bacterial communities were studied using Next Generation Sequencing (NGS) [26]. For bacterial profiling, amplification of the V1–V2 hypervariable regions of the 16S rRNA was carried out using bacterial primers (27F and 357R) followed by Ion Torrent adaptors. Forward primers were barcoded with 10 nucleotides to allow sample identification. PCR was carried out in a 25 *μ*L reaction vessel containing DNA template (1 *μ*L, 50 ng/μl), 0.2 μL reverse primer, 1 μL forward primer, 5 μL buffer (PCR Biosystems Ltd., London, UK), 0.25 μL bio HiFi polymerase (PCR Biosystems) and 17.6 μL molecular grade water. Amplification conditions for bacteria were 95°C for 1 min, then 22 cycles of 95°C for 15 s, 55°C for 15 s and 72°C for 30 s. To assess the quality of amplifications, resultant amplicons were visualized on a 1% agarose gel. PCR products were then purified using Agencourt AMpure XP beads (Beckman Coulter Inc., Fullerton, USA) and DNA concentration was determined using an Epoch Microplate Spectrophotometer fitted with a Take 3 Micro-Volume plate (BioTek, Potton, UK) to enable equimolar pooling of samples with unique barcodes.

Libraries were further purified using the EGel system with 2% agarose gel (Life Technologies Ltd., Paisley, UK). Purified libraries were assessed for quality and quantified on an Agilent 2100 Bioanalyzer with High Sensitivity DNA chip (Agilent Technologies Ltd., Stockport, UK). Library preparation for NGS sequencing was carried out using the Ion Chef system (Life Technologies UK Ltd) and the Ion PGM HiQ Chef kit, and sequencing using the Ion Torrent Personal Genome Machine (PGM) system on an Ion PGM Sequencing 316 Chips v2 BC.

Following sequencing, data were processed as previously described [26]. Briefly, sequences were trimmed to 320 bp length and mothur software (version 1.37) was used to remove low-quality sequences [27]: maximum 10 homo-polymers, Q13 average over 25 bp window and no mismatches with the primer/barcoding were allowed. Sequences were further screened for quality by discarding sequences with an expected error rate of 1 of greater using Uparse and chimera checking was performed using Uchime [28] (version 8.1). Sequences were clustered into OTUs at 97% identity using Uclust and singletons were removed. The number of reads per sample was rarefied to the sample with the lowest number of reads to obtain similar sequencing depth (10,059 sequences per animal, per sample after normalization). The Ribosomal Database Project-II Naïve Bayesian classifier [29] (Version 11.1) was used for taxonomic classification against the curated bacterial 16S rRNA database. Raw sequences were deposited at the EBI Short Read Archive form of the European Nucleotide Archive (accession number PRJEB34975).

### Volatile fatty acid measurement

Faecal samples were defrosted and diluted 1:5 w/v with distilled H_2_O (2 g sample/8 mL H_2_O) to make a faecal slurry and pH was recorded prior to the addition of 20% orthophosphoric acid (containing 20 mM 2-ethyl butyric acid as an internal standard) also at 1:5 (1 mL acid/4 mL faecal slurry) to deproteinise the samples. For VFA analyses, slurries were left for 24 h to allow sediments to settle before being syringe-filtered through a glass-fibre pre-filter (0.7 μm pore, Millipore) and a nitrocellulose membrane (0.45 μm pore; Millipore) into a glass vial and capped. VFAs were determined by gas liquid chromatography using ethyl butyric acid as the internal standard as described by [30].

### Statistical analysis

#### Phenotype data

All data was inputted into Excel and exported for statistical analysis into STATA 13.1 (Statacorp, Texas). The non-parametric Kruskal Wallis test was employed to evaluate differences in CGIT parameters and apparent digestibility between groups and years.

#### Diversity and faecal VFA analysis

Diversity indices (Inverse Simpson’s and Shannon-Wiener), species observed (S.Obs) and estimated species richness (S.Chao1) were calculated in PRIMER 7 (Primer-E, Ivybridge, UK) for all faecal samples at all time points using normalized but not square-root transformed data as recommended to reduce over-inflation of true diversity in pyrosequencing data sets [31]. Data were assessed for normality visually adopting the “ladder of powers” approach. Transformations were performed where appropriate. REML analysis (Genstat^®^ 12th edition; VSN International ltd.) was employed to assess differences in diversity, VFA concentrations and pH over time and between groups. P values were adjusted for multiple testing using the method proposed by Benjamini and Hochberg [32] to decrease the false discovery rate. Findings with P < 0.10 when applying Benjamini and Hochberg (1995) correction were regarded as statistically significant. Findings were confirmed by mixed-effect regression models (STATA 13.1, Statcorp, Texas) whereby diversity or faecal VFA concentration was the outcome variable and study day was added as an explanatory variable and polynomial terms were fitted if they improved model fit as judged by the likelihood ratio test. Pony identity was included as a random intercept. Univariate regression analysis was employed to evaluate associations between outset (mean of 3 days hay feeding) pH and faecal VFA concentrations and phenotype measurements (CGIT parameters, body fat percentage, outset/change in BCS, outset/change in BM, apparent digestibility).

#### Bacteriome data

Differences in the relative abundance of individual phyla and genera over time and between groups were investigated by REML (Genstat^®^ 12th edition; VSN International ltd.). Statistical analyses excluded those for which a relative abundance of less than 0.05% was recorded. P values were adjusted for multiple testing using the method proposed by Benjamini and Hochberg [32] to decrease the false discovery rate. Findings with P < 0.10 when applying Benjamini and Hochberg (1995) correction were regarded as statistically significant. Based on the REML analysis outputs, individual mixed effects regression models were fitted (STATA 13.1, Statacorp, Texas) for those genera with the greatest increase and decrease in relative abundance following the introduction of barley to the diet. The counts of the individual genera (square root transformed) were included as the outcome variable and study day and group (if it improved model fit as judged by the likelihood ratio test) were added as explanatory variables. Polynomial terms were fitted if they improved model fit as judged by the likelihood ratio test. Pony identity was included as a random intercept. To investigate any associations between VFA concentrations and genera abundance, separate multivariable mixed effect regression models were fitted (STATA 13.1, Statacorp, Texas) with the individual VFA as the outcome variable and the counts of the individual genera (square root transformed) included as explanatory variables and Pony identity included as a random intercept. P values were adjusted for multiple testing using the method proposed by Benjamini and Hochberg [32] to decrease the false discovery rate. Findings with P < 0.10 when applying Benjamini and Hochberg (1995) correction were regarded as statistically significant.

Principle coordinates analysis (PCoA) was employed to visually evaluate the ordination of samples at the OTU level (excluding individual OTU’s present at <0.01% relative abundance) by group and diet using a Bray-Curtis distance metric in PRIMER 7 (Primer-E, Ivybridge, UK). Permutation multivariate analysis of variance (PERMANOVA) was used to determine overall significant differences in the bacterial community and was performed in PRIMER 7. Abundance percentage data were subjected to square root transformation and Bray-Curtis distance matrices calculated. PERMANOVA was carried out using default settings with 9999 unrestricted permutations and the Monte Carlo P value was calculated. Analysis of Similarity (ANOSIM) was carried out in PRIMER 7 using the Bray-Curtis distance matrix calculated above. This analysis was used to provide a metric of the degree of divergence between communities as given by the R statistic. Community structure was investigated by non-metric Multi-Dimensional Scaling analysis of the Bray-Curtis distance matrix calculated as above using PRIMER 7.

#### Jaccard index

Stability was measured as the mean abundance of OTUs shared between study days. This was calculated for the total bacterial community at 0.01% relative abundance by calculating the Jaccard index, a coefficient of community similarity, for each animal between each of the study days using PRIMER 7.

## Results

### Body weight changes, insulin/glucose dynamics and apparent digestibility

All animals remained healthy throughout the duration of the study. During the 4-week ‘hay-washout’ phase of the study, individual animal body weight remained relatively constant (overall mean change = -0.06% ± 2.40; Control group = -0.59% ± 2.11; Aged group = 0.52% ± 2.67). The week of barley feeding resulted in overall increases in body weight (overall mean change from final hay feeding week = 2.01% ± 2.36; Control group = 2.45% ± 2.70; Aged group = 1.54% ± 1.95).

There were no significant differences in apparent digestibility of the hay or barley diet between groups or between years (S2 Table). However, within the groups, apparent digestibility (GE and DM) was significantly greater for the barley diet compared to the hay diet (p < 0.05; S2 Table). On the basis of fasted samples, all ponies were normoinsulinaemic and normoglycaemic at outset [33]. Insulin dynamics as measured by the CGIT were significantly different between the groups (S2 Table). Baseline insulin, insulin measured at 45 and 75 minutes post-infusion and area under the curve for insulin were all significantly greater in the aged compared to the control group (p < 0.05; S2 Table). There were no significant differences between groups in baseline glucose, area under the curve for glucose or time to return to baseline glucose (S2 Table).

### Coverage and diversity

Quality filtering of 16S rDNA amplicon sequences resulted in 2,466,650 high-quality sequences (320 bp long) which clustered in 12,008 different OTUs. Rarefaction curves (S1 Fig) demonstrated that sample curves had plateaued, indicating that complete sampling of these environments had been achieved. Measures of bacterial alpha diversity were not affected by either study day (p = 0.85 for Shannon, Simpson, Species observed and Chao1) or group (p = 0.82 for Shannon, Simpson, Species observed and Chao1; Table 2). Univariate regression analysis revealed no associations (p > 0.05) between outset measures of diversity (mean of 3 days on hay) and animal phenotype (CGIT parameters, body fat percentage, outset/change in BCS, outset/change in BM, apparent digestibility).

**Table 2:** Diversity and richness indices for faecal bacterial communities for the two groups across the 11 study days.

		Study day											SED			Benjamini-Hochberg P-value		
	Group	1	2	3	4	5	6	7	8	9	10	11	Day	Group	D*G	Day	Group	D*G
**Shannon**	C	5.661	5.571	5.499	5.453	5.744	5.672	5.682	5.885	5.800	5.809	5.79	0.11	0.30	0.25	0.85	0.82	0.17
	A	5.813	5.842	5.908	5.798	5.834	5.916	5.845	5.825	5.778	5.638	5.628						
**Simpson**	C	0.956	0.955	0.950	0.950	0.968	0.963	0.963	0.978	0.971	0.975	0.975	0.007	0.02	0.02	0.85	0.82	0.17
	A	0.974	0.973	0.974	0.967	0.978	0.984	0.975	0.972	0.972	0.966	0.961						
**Chao1**	C	3190	3331	3132	3446	3244	3150	3247	3073	3090	3258	2971	251.6	272	397.7	0.85	0.82	0.83
	A	3100	2985	3289	3303	3108	2897	3295	3130	3620	2775	2943						
**S.obs**	C	1729	1686	1664	1717	1777	1715	1744	1759	1734	1702	1715	68.62	135.1	132.1	0.85	0.82	0.83
	A	1652	1708	1770	1698	1638	1668	1784	1741	1714	1554	1596						

Mean diversity indices (Simpson’s and Shannon-Weiner), species observed (S.Obs) and estimated species richness (S.Chao1) for fecal samples collected over the 11 study days (final 3 days hay feeding and 8 days hay plus barley feeding) between the two groups (C, Control; A, Aged). SED: standard error of the difference.

### Faecal pH and volatile fatty acids

**Fig 1:**
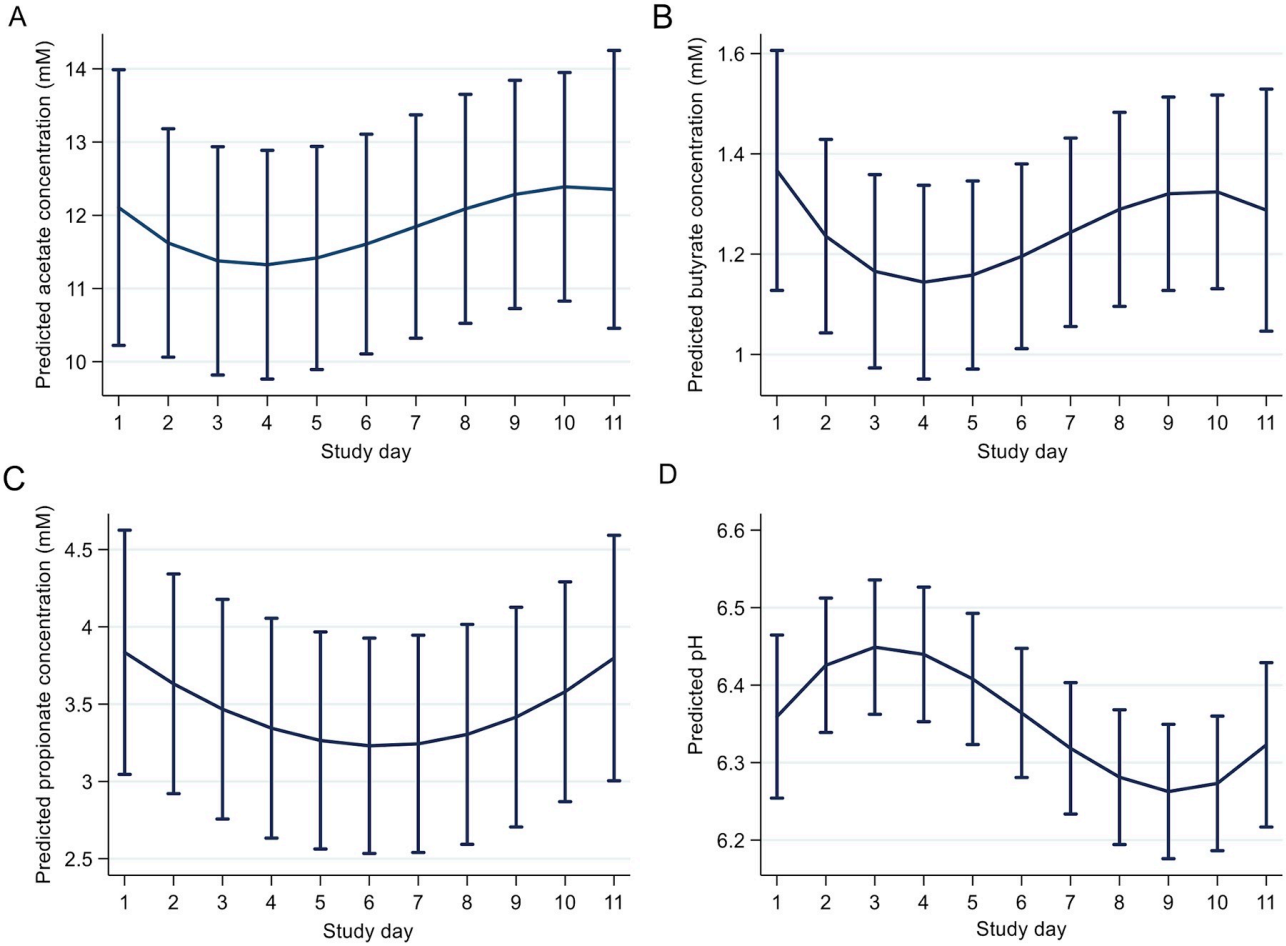
Marginal mean plots derived from mixed effect model outputs illustrating the predicted change in faecal (A) acetate, (B) butyrate, (C) propionate concentrations, and (D) pH across the study days. n = 23; error bars denote 95% confidence intervals.

Faecal VFA’s (except for BCVFA’s) and faecal pH were found to be significantly affected by study day (p < 0.05) but not group following REML analysis (Table 3). This was confirmed by mixed effects models, whereby study day was included as a cubic polynomial term (S3 Table). The intraclass correlation (ICC) estimates generated from the models ranged from 0.44 for pH to 0.65 for Propionate indicating a large portion of the variance in faecal VFA’s and pH across study days was attributable to the individual animal’s identity. Marginal mean plots depicting the predicted change in faecal VFA concentrations and pH are illustrated in Fig 1. Univariate regression analysis revealed no associations (p > 0.05) between outset faecal VFA concentration or pH (mean of 3 days on hay) and animal phenotype (CGIT parameters, body fat percentage, outset/change in BCS, outset/change in BM, apparent digestibility).

**Table 3:** pH and volatile fatty acid concentrations in faeces for the two groups across the 11 study days.

		Study day											SED			Benjamini-Hochberg P-value		
	Group	1	2	3	4	5	6	7	8	9	10	11	Day	Group	D*G	Day	Group	D*G
**Acetate (mM)**	C	10.35	12.21	13.33	12.10	10.81	11.18	11.19	11.92	15.20	11.84	11.48	1.07	1.50	1.81	0.03	0.87	0.77
	A	12.26	12.00	12.10	12.13	8.88	10.05	11.22	12.33	12.54	14.12	11.41						
**Butyrate (mM)**	C	1.17	1.19	1.28	1.19	1.01	1.11	1.06	1.33	1.51	1.11	1.11	0.15	0.18	0.24	0.06	0.64	0.58
	A	1.41	1.32	1.43	1.26	0.96	1.09	1.20	1.36	1.47	1.65	1.29						
**Propionate (mM)**	C	3.28	3.45	3.77	3.42	2.87	2.99	2.71	3.02	3.60	2.95	3.19	0.34	0.72	0.68	0.00	0.64	0.58
	A	3.80	4.21	4.34	3.56	2.59	2.95	3.16	3.85	4.16	4.90	3.74						
**BCVFA (mM)**	C	1.22	1.51	1.32	1.11	1.07	1.21	1.26	1.38	1.69	1.10	1.06	0.41	0.33	0.62	0.63	0.57	0.58
	A	1.73	1.56	1.56	3.38	1.61	1.40	1.60	1.44	1.51	1.84	1.39						
**pH**	C	6.32	6.43	6.32	6.43	6.40	6.44	6.25	6.17	6.16	6.12	6.35	0.06	0.08	0.10	0.00	0.57	0.58
	A	6.50	6.36	6.34	6.56	6.42	6.48	6.40	6.38	6.32	6.29	6.42						

Faecal pH and VFA concentrations for samples collected over the 11 study days (final 3 days hay feeding and 8 days hay plus barley feeding) between the two groups (C, Control; A, Aged). BCVFA = Branched chain volatile fatty acids. SED: standard error of the difference.

### Phyla

**Fig 2:**
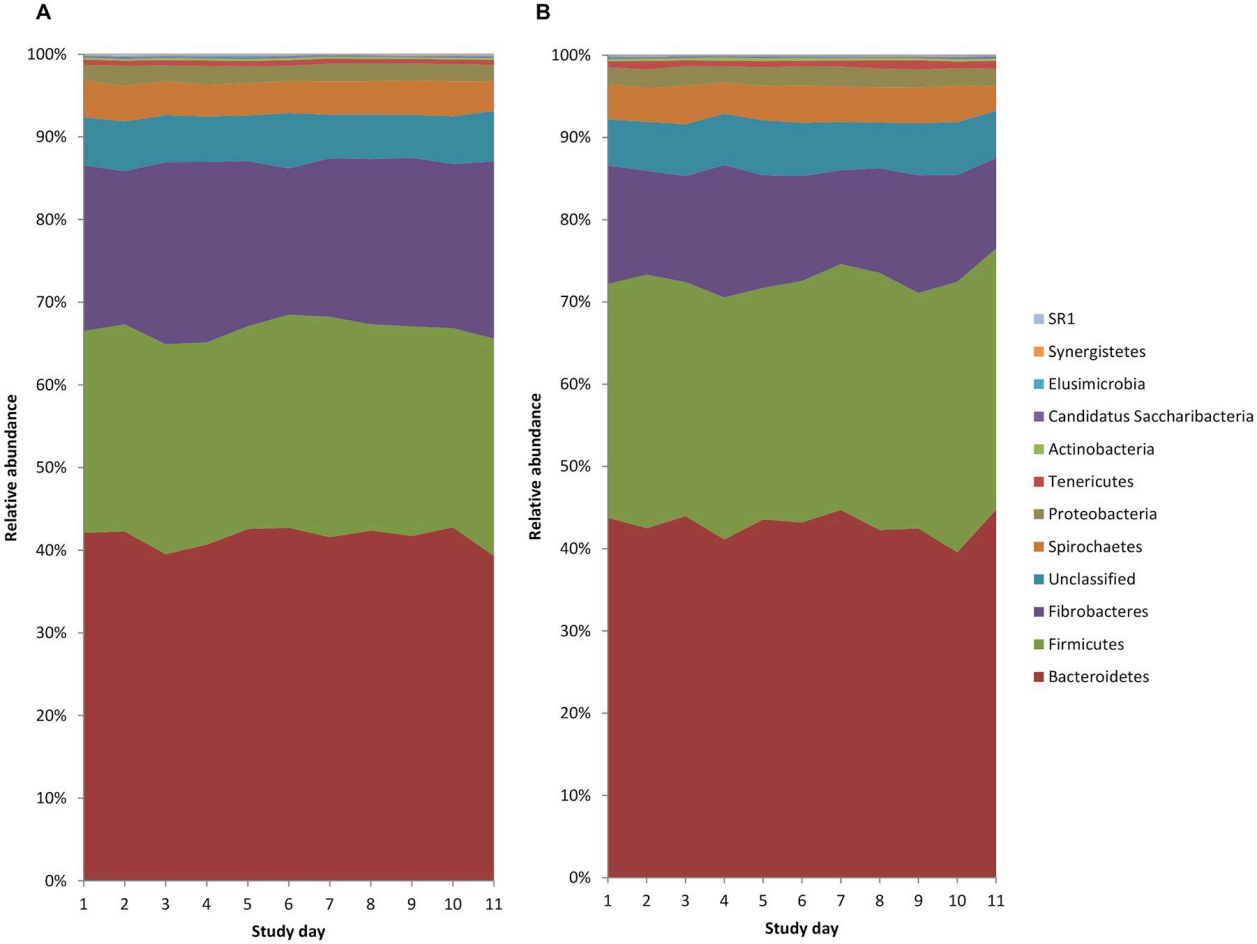
Relative abundance of bacterial phyla (including all phyla at > 0.05% relative abundance) in (A) Control (n = 12) and (B) Aged (n = 11) animals across the study days.

Across all study days, the most abundant phyum were the *Bacteroidetes*, followed by the *Firmicutes* and *Fibrobactres* (Fig 2). There were no significant differences in the relative abundance of individual phylum between the control and aged groups (p > 0.05; Table 4) on any of the study days; however there was a significant increase in the relative abundance of *Candidatus Saccharibacteria* and *Firmicutes* and a corresponding reduction in the relative abundance of *Fibrobactres* across the study days, following the addition of barley to the diet (S4 Table).

**Table 4:** The effect of study day and group on the relative abundance of bacterial phyla.

	SED			Benjamini-Hochberg P-value		
	Group	Day	G*D	Group	Day	G*D
*Actinobacteria*	0.04	0.03	0.05	0.65	0.42	0.37
*Bacteroidetes*	0.16	0.11	0.18	0.65	0.32	0.60
*Candidatus Saccharibacteria*	0.04	0.03	0.05	0.87	0.08	0.37
*Elusimicrobia*	0.04	0.04	0.06	0.92	0.75	0.72
*Fibrobacteres*	0.56	0.19	0.45	0.65	0.04	0.77
*Firmicutes*	0.26	0.11	0.23	0.65	0.01	0.60
*Proteobacteria*	0.13	0.08	0.14	0.91	0.37	0.39
*Spirochaetes*	0.11	0.07	0.12	0.91	0.56	0.77
*SR1*	0.06	0.03	0.06	0.87	0.42	0.93
*Synergistetes*	0.02	0.02	0.03	0.87	0.42	0.37
*Tenericutes*	0.11	0.04	0.10	0.92	0.42	0.37
*unclassified*	0.17	0.09	0.17	0.91	0.76	0.93

SED: standard error of the difference.

### Genera

**Fig 3:**
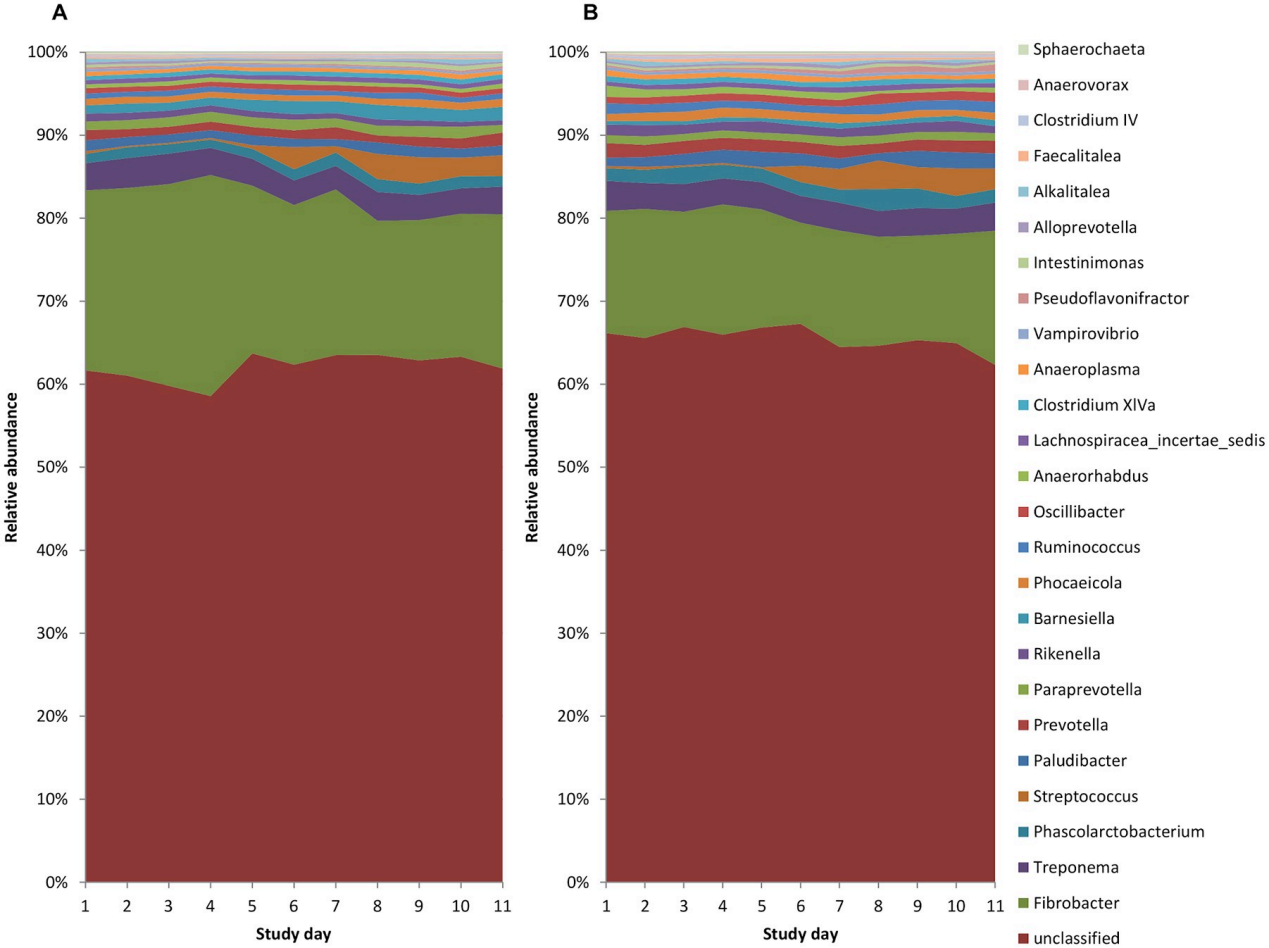
Relative abundance of bacterial genera (including all genera at > 0.20% relative abundance) in (A) Control (n = 12) and (B) Aged (n = 11) animals across the study days.

Although many OTU’s were unclassified at a genus level (Fig 3), the overall relative abundance of *Barnesiella* was found to be significantly greater in the control compared to the aged group (Control mean relative abundance = 1.31% ± 0.10 (SD), Aged mean relative abundance = 0.52%, ± 0.29 p < 0.05; Table 5). Additionally, the relative abundance of 9 individual genera changed during the week of barley feeding (S4 Table). Over the course of the study, the relative abundance of *Streptococcus* demonstrated the largest increase, whilst *Fibrobacter* correspondingly showed the largest decrease in relative abundance, following the introduction of barley to the diet (S4 Table). This was confirmed using mixed models, whereby study day was included as a cubic polynomial term (S5 Table). As for the VFA and pH data, the intraclass correlation estimates generated from the models identified a significant portion of the variation in *Fibrobacter* and *Streptococcus* counts was due to individual animal (S5 Table). There were no associations identified between VFA concentrations and the relative abundance of any genera (adjusted p-values > 0.10; S6 Table).

**Table 5:** The effect of study day and group on the relative abundance of bacterial genera.

	SED			Benjamini-Hochberg P-value		
	Group	Day	D*G	Group	Day	D*G
*Fibrobacter*	0.57	0.19	0.46	0.79	0.06	0.89
*Paludibacter*	0.24	0.08	0.19	0.84	0.71	0.73
*unclassified*	0.09	0.03	0.07	0.84	0.71	0.82
*Oscillibacter*	0.07	0.04	0.07	0.47	0.06	0.57
*Lachnospiracea_incertae_sedis*	0.08	0.04	0.08	0.84	0.34	0.89
*Mobilitalea*	0.04	0.02	0.04	0.87	0.81	0.57
*Phocaeicola*	0.13	0.06	0.11	0.84	0.89	0.62
*Phascolarctobacterium*	0.06	0.12	0.13	0.75	0.02	0.57
*Saccharibacteria_genera_incertae_sedis*	0.06	0.03	0.06	0.84	0.52	0.93
*Sporobacter*	0.08	0.03	0.07	0.84	0.47	0.40
*Mogibacterium*	0.04	0.03	0.04	0.91	0.81	0.89
*Ruminococcus*	0.10	0.06	0.11	0.47	0.52	0.90
*Sphaerochaeta*	0.04	0.03	0.05	0.84	0.09	0.57
*Lactobacillus*	0.03	0.02	0.04	0.84	0.02	0.89
*Faecalicoccus*	0.06	0.03	0.06	0.87	0.81	0.98
*Treponema*	0.11	0.07	0.12	0.87	0.70	0.89
*Anaeroplasma*	0.10	0.05	0.10	0.84	0.74	0.73
*Paraprevotella*	0.12	0.04	0.10	0.84	0.37	0.62
*Lachnobacterium*	0.06	0.03	0.06	0.90	0.71	0.98
*Pseudoflavonifractor*	0.08	0.07	0.11	0.47	0.14	0.73
*Clostridium IV*	0.05	0.03	0.05	0.62	0.06	0.78
*Coprobacter*	0.12	0.04	0.10	0.84	0.66	0.73
*Rikenella*	0.10	0.06	0.11	0.47	0.19	0.98
*Intestinimonas*	0.06	0.04	0.07	0.84	0.04	0.73
*Anaerovorax*	0.05	0.02	0.04	0.84	0.60	0.78
*Alloprevotella*	0.08	0.04	0.08	0.79	0.34	0.89
*Clostridium XlVa*	0.09	0.04	0.08	0.84	0.99	0.73
*Prevotella*	0.11	0.06	0.12	0.79	0.29	0.62
*Butyricicoccus*	0.04	0.03	0.04	0.84	0.19	0.73
*Streptococcus*	0.27	0.19	0.32	0.84	0.02	0.98
*Blautia*	0.03	0.02	0.04	0.84	0.54	0.73
*Barnesiella*	0.10	0.09	0.15	0.05	0.19	0.93
*SR1_genera_incertae_sedis*	0.03	0.02	0.04	0.84	0.42	0.73
*Anaerobacterium*	0.02	0.02	0.03	0.84	0.89	0.89
*Candidatus Endomicrobium*	0.04	0.03	0.05	0.84	0.91	0.73
*Acetanaerobacterium*	0.05	0.03	0.05	0.47	0.52	0.89
*Parvibacter*	0.04	0.02	0.04	0.84	0.29	0.10
*Papillibacter*	0.02	0.02	0.03	0.84	0.42	0.40
*Alkalitalea*	0.14	0.07	0.14	0.84	0.19	0.76
*Catabacter*	0.04	0.04	0.07	0.84	0.70	0.57
*Ethanoligenens*	0.06	0.04	0.06	0.84	0.81	0.73
*Vampirovibrio*	0.24	0.10	0.22	0.84	0.80	0.73
*Butyrivibrio*	0.10	0.07	0.12	0.79	0.89	0.73
*Faecalitalea*	0.08	0.04	0.08	0.47	0.63	0.73
*Christensenella*	0.04	0.03	0.05	0.79	0.99	0.88
*Anaerorhabdus*	0.16	0.09	0.16	0.84	0.81	0.89
*Ureaplasma*	0.04	0.03	0.05	0.84	0.29	0.93
*Anaerotruncus*	0.05	0.02	0.05	0.87	0.60	0.73
*Pyramidobacter*	0.02	0.02	0.03	0.84	0.52	0.05
*Asteroleplasma*	0.05	0.03	0.05	0.47	0.99	0.89
*Anaerocella*	0.05	0.02	0.05	0.89	0.09	0.78
*Catenibacterium*	0.03	0.03	0.05	0.84	0.71	0.57

SED: standard error of the difference.

### Streptococcus

**Fig 4:**
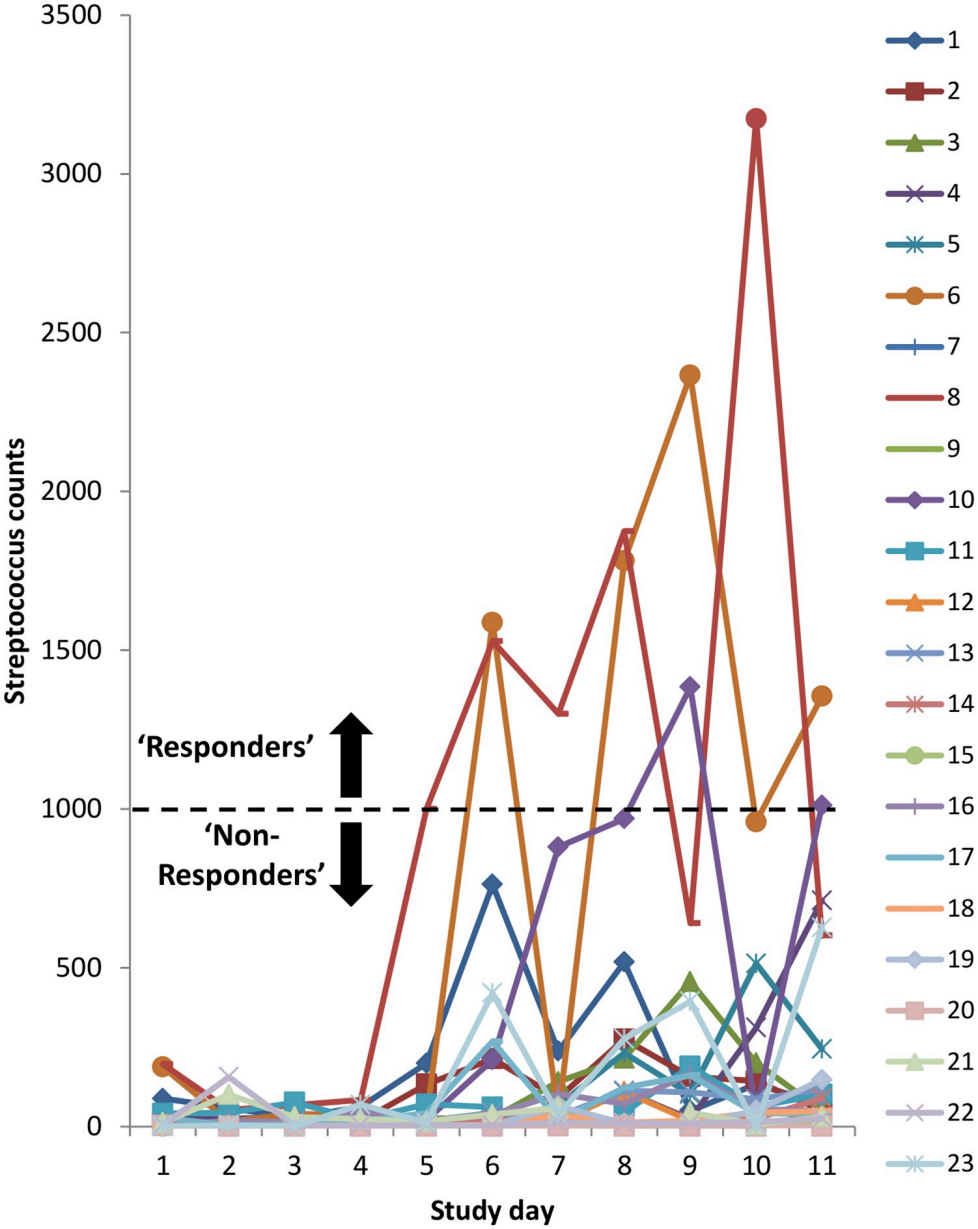
Raw *Streptococcus* count data for individual animals across the study days. Three animals displayed elevated *Streptococcus* counts during barley feeding (plotting above dashed line) and were classified as ‘responders’ (PonyID 6, 8 and 10).

Upon closer inspection of the raw *Streptococcus* count data, the individual variation in response to barley feeding became clear (Fig 4). The data demonstrates that 3 animals (PonyID 6, 8 and 10) displayed a greater *Streptococcus* response (raw counts exceeding 1000) to barley feeding (‘responders’) compared to the other animals (‘non-responders’). Logistic regression analysis using ‘responder’ as the outcome variable revealed no associations (p > 0.05) for any outset phenotype measurement (CGIT parameters, body fat percentage, outset/change in BCS, and outset/change in BM), outset diversity, pH or VFA concentrations (S7 Table).

Whilst there was no change in bacterial diversity across the study days when all animals were included in the analysis, data was re-analysed separately for ‘responders’ and ‘non-responders’. Significant negative associations were identified between diversity and study day in those animals classified as responders (p < 0.05; Table 6).

**Table 6:** Associations between bacterial richness and diversity and study day in those animals classified as ‘responders’ (n = 3) based on raw *Streptococcus* count data.

Outcome variable	Explanatory variable	Coefficient	P-value	95% confidence interval
**Shannon**	Study day	-0.09	<0.001	-0.14 to -0.05
	Baseline	6.27	<0.001	5.96 to 6.58
**Random effects parameter: Pony ID**	Variance (baseline)	0.003		1.77e-08 to 374.62
	Variance (residual)	0.17		0.10 to 0.29
**Simpson**	Study day	-0.002	0.001	-0.004 to -0.001
	Baseline	0.99	<0.001	0.98 to 1.00
**Random effects parameter: Pony ID**	Variance (baseline)	4.55e-27		7.83e-49 to 0.002
	Variance (residual)	0.001		0.0001 to 0.0003
**Chao1**	Study day	-87.90	0.07	-181.75 to 5.95
	Baseline	3427.28	<0.001	2786.23 to 4068.33
**Random effects parameter: Pony ID**	Variance (baseline)	2.22e-19		0
	Variance (residual)	754286.70		462100.10 to 1231223.0
**Sobs**	Study day	-56.98	<0.001	-85.80 to -28.17
**Random effects parameter: Pony ID**	Baseline	1963.24	<0.001	1745.64 to 2180.85
	Variance (baseline)	6720.15		261.82 to 172489.00
	Variance (residual)	71080.07		42472.36 to 118956.80

### Core bacterial community

The core bacterial community was defined as bacteria present in all samples analysed in the current study at a relative abundance of 0.1% or greater. When all the samples were considered, only a single OTU made up the core bacterial community, belonging to the *Prevotella* genus and accounting for on average 0.56% of total sequences recovered (Control hay 0.52% ± 0.57, Control barley 0.48% ± 0.39, Aged hay 0.75% ± 0.34, Aged barley 0.58% ± 0.42; S8 Table). Similarly, when barley-only samples were considered, only a single OTU made up the core bacterial community (the same OTU as for all samples), accounting for an average 0.52% of total sequences recovered (Control group 0.48% ± 0.32; Aged group 0.57% ± 0.44; S8 Table). When the hay-only samples were considered, a total of 9 OTU’s made up the core bacterial community, accounting for on average 3.56% of total sequences recovered (Control 3.32% ± 0.23; Aged 3.80% ± 0.22; S8 Table).

### Bacterial community structure and stability

**Fig 5:**
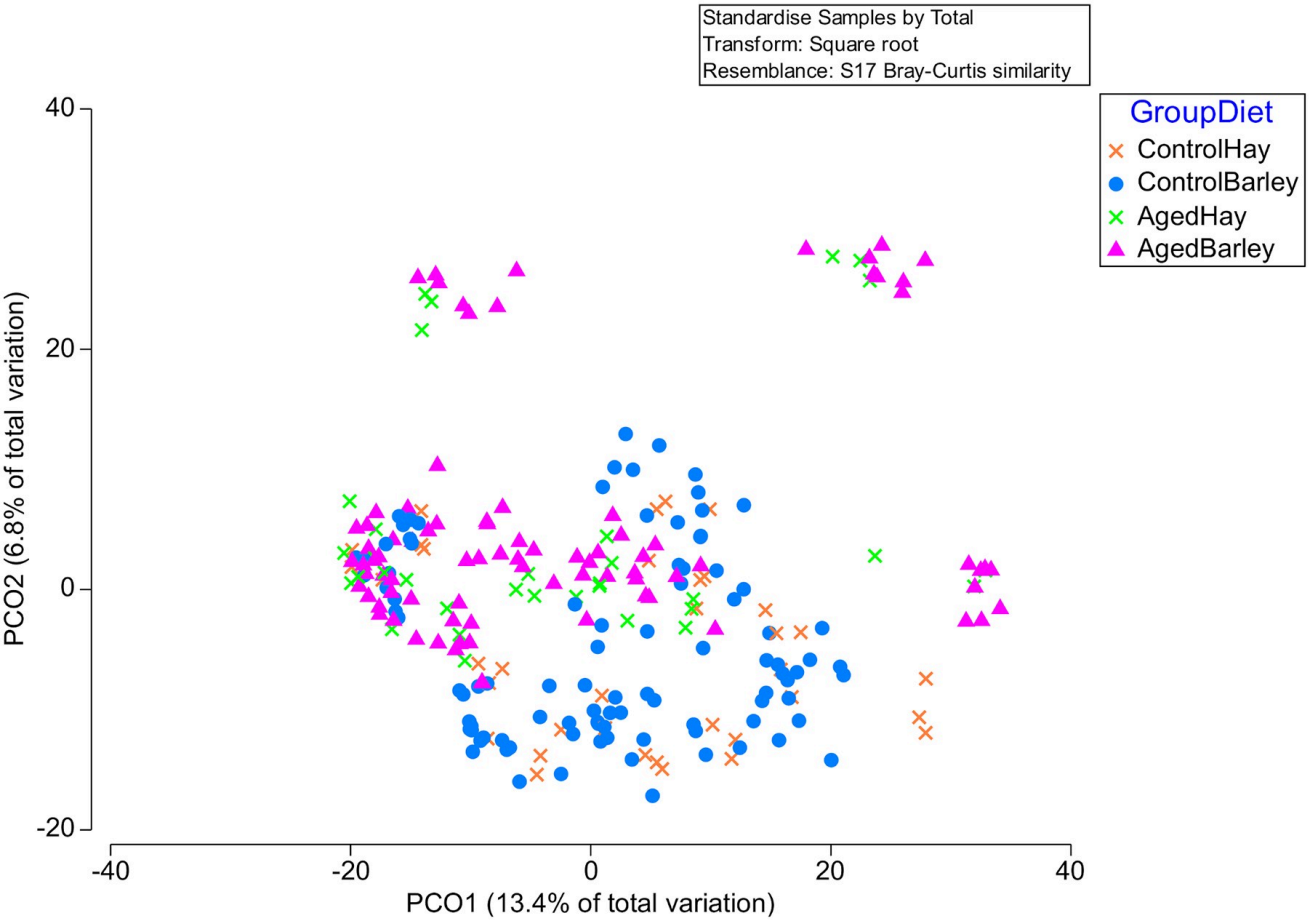
Principle coordinate analysis of the bacterial OTU data (excluding those OTU’s present at <0.01% relative abundance) by group and diet.

Principal coordinates analysis, PERMANOVA and ANOSIM (S9 Table. Fig 5) suggested that the bacterial community clustered by group (p = 0.01, R = 0.15) but not diet and there was no group x diet interaction. Non-metric Multi-Dimensional Scaling analysis showed no clear clustering by group or diet (S2 Fig).

As in our previous study we used the Jaccard index to determine the stability of abundance of OTUs abundance shared between days. The Jaccard coefficient measures similarity between finite sample sets, and is defined as the size of the intersection divided by the size of the union of the sample set [34]. Bacterial community stability was not affected by either study day or group (S3 Fig); however variation across study days was found to be greater in the aged compared to the control group. Stability was also evaluated between day 3 (final day on hay) and day 11 (final day on barley) to ascertain the extent of perturbation of the faecal bacteriome resulting from barley feeding. Average stability values were found to be 0.67 ± 0.69 and 0.64 ± 0.92 for the Control and Aged groups, respectively.

## Discussion

The current study describes changes in the faecal bacteriome of aged and control ponies associated with the transition and adaptation from a hay-based diet to a high-starch diet. It has previously been shown that aged horses display a reduction in species richness and bacterial diversity when fed a range of different diets [9] and it was hypothesised that aged ponies in the current study would also demonstrate a reduction in bacterial diversity, and a more marked perturbation of the faecal microflora compared to control animals following the incorporation of starch into the diet.

Conversely, despite animals in the current study being of the same breed and sex, the impact of age on the faecal bacteriome composition was not as profound as expected. There were no differences in bacterial diversity between the aged and control groups, and only a single genus was found to be affected by age. *Barnesiella*, a propionate producer belonging to the *Bacteroidetes* phylum was found to be significantly more abundant in the control vs. the aged animals in the current study. The faecal microbiome of aged mice was similarly found to possess a lower abundance of *Barnesiella* compared to control animals, which was suggested may contribute to the increased allergic inflammation observed in aged mice [35]. Additionally, in the present study, the stability (Jaccard index) of the bacterial community was not found to differ between control and aged ponies, although there appeared to be a greater degree of variation between individual animals within the aged group. The stability values obtained when comparing the final day on hay to the final day of barley feeding were comparable to those obtained by Dougal et al. (2017) when evaluating the faecal microbiome of a group of horses and ponies maintained on a fibre-based diet across a 6-week period.

The aged animals in the current study were recruited based on their chronological age, and it may be that these aged animals were more similar to the control group in terms of their biological age, a concept in age-based research to account for differing rates of senescence [36]. In agreement with this, it has previously been shown in humans that biological (measured by an index of frailty) as opposed to chronological age is strongly associated with aspects of the gastrointestinal microbiota, including measures of diversity [37]. Furthermore, although chronologically similar in age, Dougal et al. (2014) evaluated horses as opposed to ponies, which may be experiencing senescent changes earlier than ponies.

Whilst there was a lack of group effect, the addition of barley to the diet led to compositional changes at the phyla level including a reduction in the relative abundance of *Fibrobactres* and an increase in the relative abundance of *Firmicutes* phylum. This was unsurprising since reductions in this fibrolytic group of bacteria that play a major role in fibre fermentation illustrates a shift in microbiome composition towards more acid-tolerant species specialised in starch fermentation, such as *Streptococcus* and *Lactobacillus*, both belonging to the *Firmicutes* phylum [38]. Compositional changes at the genera level were dominated by increases in the relative abundance of *Streptococcus* following the addition of barley to the diet. This is in agreement with several other studies evaluating amylolytic bacterial populations including *Streptococcus* in horses following supplementation of the diet with starch using both culture-independent [39,40] and culture-dependent techniques [8,10]. However, upon closer inspection of the raw count data for *Streptococcus* in the current study, it became apparent that there was considerable variation between individual animals. Large inter-animal variability has been a common finding amongst numerous other equine microbiome studies [3,8–10]. The relative abundance of *Lactobacillus* also increased following the addition of barley in the present study, although it was present at a much lower abundance relative to *Streptococcus*, and was not accompanied by the same degree of inter-individual animal variation.

Dynamic modelling of the equine ceacal microbiota has shown that *Streptococcus* promoted its own growth following the introduction of barley to the diet [39] and may indicate that those ‘responders’ in the current study were unable to maintain a favourable environment for the growth of lactate-utilising bacterial populations, which in turn allowed the overgrowth of *Streptococcus*. Increases in the abundance of Streptococcal species in the equine caecum has been identified prior to the onset of oligofructose-induced laminitis [41], and has been shown to contribute to the development of ruminal acidosis, which can cause major digestive upset amongst cattle and sheep [42,43]. Although animals in the current study remained healthy throughout, it could be suggested that the large variation in *Streptococcus* response reflects variation in individual susceptibility to gastrointestinal disturbances and other metabolic disorders. The application of additional ‘omics’ technologies such as metagenomics and metatranscriptomics to evaluate the functional activity of the bacterial species may also give further insight into the reasoning behind the individual variation to dietary responses. This approach is widely used to help answer key questions in the study of the rumen microbiome [44,45].

The ability to identify those ‘high risk’ animals who may display an elevated *Streptococcus* response prior to the introduction of dietary starch would be advantageous in aiding dietary management strategies. The extent of pre-caecal starch digestion in horses is affected by multiple factors including botanical origin, level of intake and the degree of feed processing [13]. Starch in the current study was fed in the form of micronized steam-flaked barley, and was gradually introduced over a 4-day period to reach a maximum rate of 2g starch per kg BM fed in a single morning meal, the maximum daily recommended level to avoid an overload of undigested starch reaching the hindgut [13,15]. Thermo-mechanically treated barley processing has been found to have greater pre-caecal starch digestibility compared to whole grain barley [46], and providing barley in this form may also limit the reduction in fibre degradation in the hindgut [14].

Concentrations of VFA and faecal pH were significantly altered by the incorporation of barley into the diet in the current study. Faecal pH was reduced following the addition of starch to the diet, with levels appearing to return towards baseline values by the final day of sampling, suggesting an adaptation to the diet. In agreement with this, Grimm et al., (2017) concluded that the hindgut microbial ecosystem adapted to a diet of hay and barley (> 2g starch/kg BM daily) 10 days following transition from a hay-only diet, as minimal changes in ecosystem parameters were identified between days 10 and 20 on the hay and barley diet [10]. The addition of starch into the diet has been shown to be associated with increases in caecal propionate concentration, a consequence of the proliferation of amylolytic bacterial species populating the hindgut [10,39]. However, changes in faecal VFA concentrations following high starch diets have not been as consistent [10,47], which may be reflective of differences in the absorption of VFA’s along the hindgut.

In the current study, no associations were identified between measures of outset animal phenotype and the *Streptococcus* responders. However, although outset measures of α-diversity were not different between ‘responders’ and ‘non-responders’ based on the raw count *Streptococcus* data, they were found to decrease over the course of the trial in ‘responders’, a finding that was masked when all animals were considered collectively. Due to the compositional nature of the bacterial populations inhabiting the gastrointestinal tract, the rapid growth of *Streptococcus* bacteria will have occurred at the expense of other less competitive bacterial species, thereby reducing the overall bacterial diversity in those ‘responders’.

Measures of diversity and richness are used to describe microbial ecosystems within the gastrointestinal tract, with species rich and diverse communities considered to be more resilient to disturbance [48,49]. Fibre-rich diets promote microbial richness and diversity, and although some studies have demonstrated a reduction in measures of diversity following the introduction of starch into the diet of horses [9,40], others have shown no change [39,50]. Discrepancies between studies are likely due to different study designs, dietary compositions and sampling sites. The current study utilised faecal samples for subsequent microbial analysis, which have previously been found to possess a more similar microbiota composition to the distal hindgut compared to the proximal hindgut region [51]. More recently however, concentrations of cellulolytic, amylolytic and lactate-utilising bacteria were found to be significantly correlated between the caecal and faecal samples and between the right-ventral colon and faecal samples, suggesting that elements of the faecal microbial ecosystem are representative of the proximal hindgut ecosystem during dietary change [10].

Evaluation of the core microbial population in the current study identified only a single OTU present in every sample at an abundance of at least 0.1%. The core bacteriome population in the horse has been consistently shown to be significantly smaller than in other species [3,4,52], which may partly explain why horses and ponies are more susceptible to gastrointestinal disorders. Data from the current study identified only a single OTU constituting the core population, growing only marginally when only the hay samples were considered. Similarly, the core bacteriome was found to be smaller in horses fed a carbohydrate-supplemented diet compared to a hay-only diet, albeit still larger than that found in the current study [9].

## Conclusions

In conclusion, the current study has described changes in the faecal bacteriome composition of ponies associated with the transition from a hay-based to a high starch diet. Although little effect of age was identified in terms of bacterial diversity and bacteriome composition, significant inter-individual variation in the *Streptococcus* response to barley feeding was a key finding and associated with a reduction in bacterial diversity over the course of the barley feeding period in those animals with an elevated *Streptococcus* response. Whilst the current study utilised a metataxonomic approach to describing the bacteriome composition, future studies may be directed towards understanding bacterial function during dietary changes.

## Supporting information

S1 Table Composition of the diets used in the current study. (DOCX)

S2 Table CGIT and apparent digestibility data for the animals in the control (n = 12) and aged (n = 11) groups. Data presented are mean ± SD for the two groups. Outcome values for the combined glucose-insulin tolerance test (CGIT) were fasted baseline values for plasma glucose and insulin concentrations, insulin concentrations 45 and 75 minutes post-infusion, the areas under the curves (AUC) for insulin and glucose and the time taken for glucose concentrations to return to baseline values. The apparent digestibilities of gross energy (GE) and dry matter (DM) are also shown. *denotes significant difference compared to Control group. Different superscripts within rows indicate significant between-diet differences.
(DOCX)

S3 Table Associations between volatile fatty acids, pH and study day. Mixed-effects linear regression models (random effects: Pony ID) were built with volatile fatty acids and pH as the outcome variables and study day as a cubic polynomial as the explanatory variable. An unstructured covariance matrix was employed for the random effects. Coefficients and the intraclass correlation coefficients are presented ± 95% confidence intervals (CI’s).
(DOCX)

S4 Table The relative abundance of bacterial phyla and genera significantly affected by study day. Relative abundance of bacterial phyla and genera across the 11 study days identified to be significantly altered by study day following REML analysis. SED = standard error of the difference.
(DOCX)

S5 Table Associations between *Streptococcus*, *Fibrobacter* and study day. Mixed-effects linear regression models (random effects: Pony ID) were built with either *Streptococcus* or *Fibrobacter* counts (square-root transformed) as the outcome variables and study day as a cubic polynomial as the explanatory variable. An unstructured covariance matrix was employed for the random effects. Coefficients and the intraclass correlation coefficients are presented ± 95% confidence intervals (CI’s).
(DOCX)

S6 Table Associations between VFA concentrations and the relative abundance of bacterial genera. Mixed-effects multivariable regression models were built in with the individual VFA as the outcome variable and the counts of the individual genera (square root transformed) included as explanatory variables and pony identity included as a random intercept. Coefficients ± 95% confidence intervals (95% CI) and p-values (following adjustment for multiple testing using the method proposed by Benjamini and Hochberg to decrease the false discovery rate) are presented. BCVFA: branched chain volatile fatty acids.
(DOCX)

S7 Table Outset phenotype parameters in ‘responders’ or ‘non-responders’. Separate logistic regression models were built with the binary variable ‘responder’ (based on raw *Streptococcus* counts) as the outcome variable and individual phenotype parameters (including CGIT parameters, outset bacterial diversity and outset faecal VFA/pH) as the explanatory variables.
(DOCX)

S8 Table Core OTU population. (DOCX)

S9 Table Effect of group (control and aged) and diet on the structure of the bacterial communities in the faeces. Summary of ANOSIM and PERMANOVA outputs. Significant P-values for PERMANOVA (p < 0.05) are highlighted. ANOSIM R-values indicate the degree of separation between samples (0 = very similar; 1 = highly dissimilar), with significant R-values (with p < 0.05) shown in bold.
(DOCX)

S1 Fig Rarefaction curves. (PDF)

S2 Fig Non-metric Multi-Dimensional scaling plot. (PDF)

S3 Fig Jaccard index between the individual study days for the control and aged groups. (PDF)
