# Supplementary material for: Effect of age and the individual on the gastrointestinal bacteriome of ponies fed a high-starch diet
Source: PLoS One. 2020 May 8;15(5):e0232689. doi: 10.1371/journal.pone.0232689 (PMC7209120; doi:10.1371/journal.pone.0232689)
Supplement: S1 Table — (DOCX) [file pone.0232689.s001.docx]

**Table S1: Composition of the diets used in the current study**

|  | **Hay** | **Barley** | **Balancer** |
| --- | --- | --- | --- |
| **Gross energy (MJ/kg DM)** | 18.9 | 18.3 | 18.0 |
| **Ash (% DM)** | 4.0 | 1.7 | 14.0 |
| **Crude Protein (% DM)** | 8.1 | 11.9 | 21.1 |
| **Acid detergent fibre (% DM)** | 41.2 | 5.4 | 14.3 |
| **Neutral detergent fibre (% DM)** | 64.7 | 16.2 | 31.7 |
| **Starch (% DM)** | 0.6 | 60.0 | 10.5 |
| **Water soluble carbohydrates (% DM)** | 15.6 | 2.5 | 12.5 |
