# Supplementary material for: Effect of age and the individual on the gastrointestinal bacteriome of ponies fed a high-starch diet
Source: PLoS One. 2020 May 8;15(5):e0232689. doi: 10.1371/journal.pone.0232689 (PMC7209120; doi:10.1371/journal.pone.0232689)
Supplement: S2 Table — Data presented are mean ± SD for the two groups. Outcome values for the combined glucose-insulin tolerance test (CGIT) were fasted baseline values for plasma glucose and insulin concentrations, insulin concentrations 45 and 75 minutes post-infusion, the areas under the curves (AUC) for insulin and glucose and the time taken for glucose concentrations to return to baseline values. The apparent digestibilities of gross energy (GE) and dry matter (DM) are also shown. *denotes significant difference compared to Control group. Different superscripts within rows indicate significant between-diet differences. (DOCX) [file pone.0232689.s002.docx]

**Table S2: CGIT and apparent digestibility data for the animals in the Control (n = 12) and Aged (n = 11) groups.**

|  | **Control** | **Aged** |
| --- | --- | --- |
| **Baseline insulin (µIU/ml)** | 2.13 ± 0.42 | 8.17 ± 8.22* |
| **Insulin time 45 (µIU/ml)** | 47.6 ± 29.92 | 114.70 ± 79.27* |
| **Insulin time 75 (µIU/ml)** | 12.35 ± 7.17 | 53.86 ± 58.99* |
| **AUC insulin (µIU/ml/min)** | 2018.03 ± 1231.08 | 5292.86 ± 3849.65* |
| **Baseline glucose (mmol/L)** | 5.23 ± 0.57 | 5.48 ± 0.59 |
| **AUC glucose (mmol/L/min)** | 774.60 ± 121.13 | 914.18 ± 178.11 |
| **Return to baseline glucose concentration (minutes)** | 50 ± 24.49 | 79.09 ± 50.09 |
| **DM digestibility (%)** | Hay: 51.51 ± 3.54^a^  Barley: 57.15 ± 4.66^b^ | Hay: 52.52 ± 3.15^a^  Barley: 59.71 ± 6.84^b^ |
| **GE digestibility (%)** | Hay: 50.08 ± 4.66^a^  Barley: 55.42 ± 5.65^b^ | Hay: 51.15 ± 3.11^a^  Barley: 59.75 ± 5.98^b^ |

Data presented are mean ± SD for the two groups. Outcome values for the combined glucose-insulin tolerance test (CGIT) were fasted baseline values for plasma glucose and insulin concentrations, insulin concentrations 45 and 75 minutes post-infusion, the areas under the curves (AUC) for insulin and glucose and the time taken for glucose concentrations to return to baseline values. The apparent digestibilities of gross energy (GE) and dry matter (DM) are also shown. *denotes significant difference compared to Control group. Different superscripts within rows indicate significant between-diet differences.
