# Supplementary material for: Effect of age and the individual on the gastrointestinal bacteriome of ponies fed a high-starch diet
Source: PLoS One. 2020 May 8;15(5):e0232689. doi: 10.1371/journal.pone.0232689 (PMC7209120; doi:10.1371/journal.pone.0232689)
Supplement: S3 Table — Mixed-effects linear regression models (random effects: Pony ID) were built with volatile fatty acids and pH as the outcome variables and study day as a cubic polynomial as the explanatory variable. An unstructured covariance matrix was employed for the random effects. Coefficients and the intraclass correlation coefficients are presented ± 95% confidence intervals (CI’s). (DOCX) [file pone.0232689.s003.docx]

**Table S3:** **Associations between volatile fatty acids, pH and study day.**

| **Outcome variable** | **Explanatory variable** | **Coefficient** | **P-value** | **95% confidence interval** |
| --- | --- | --- | --- | --- |
| **Acetate** | Study day | -0.93 | 0.32 | -2.75 to 0.89 |
|  | Study day^2^ | 0.17 | 0.34 | -0.18 to 0.51 |
|  | Study day^3^ | -0.01 | 0.42 | -0.03 to 0.01 |
|  | Baseline | 12.88 | <0.001 | 9.92 to 15.83 |
| **Random effects parameter: Pony ID** | Variance (baseline) | 10.76 |  | 5.60 to 20.33 |
|  | Variance (residual) | 1.25 |  | 10.92 to 15.83 |
| **Intraclass correlation** | 0.45 |  |  | 0.29 to 0.62 |
| **Propionate** | Study day | -0.26 | 0.39 | -0.86 to 0.34 |
|  | Study day^2^ | 0.02 | 0.75 | -0.09 to 0.13 |
|  | Study day^3^ | 0.003 | 0.92 | -0.01 to 0.01 |
|  | Baseline | 4.08 | <0.001 | 2.99 to 5.17 |
| **Random effects parameter: Pony ID** | Variance (baseline) | 2.61 |  | 1.42 to 4.79 |
|  | Variance (residual) | 1.41 |  | 1.17 to 1.70 |
| **Intraclass correlation** | 0.65 |  |  | 0.49 to 0.78 |
| **Butyrate** | Study day | -0.25 | 0.05 | -0.90 to -0.001 |
|  | Study day^2^ | 0.04 | 0.07 | -0.003 to 0.09 |
|  | Study day^3^ | -0.002 | 0.11 | -0.005 to 0.0004 |
|  | Baseline | 1.57 | <0.001 | 1.18 to 1.96 |
| **Random effects parameter: Pony ID** | Variance (baseline) | 0.15 |  | 0.078 to 0.30 |
|  | Variance (residual) | 0.24 |  | 0.20 to 0.29 |
| **Intraclass correlation** | 0.39 |  |  | 0.24 to 0.56 |
| **pH** | Study day | 0.15 | 0.004 | 0.05 to 0.25 |
|  | Study day^2^ | -0.03 | 0.001 | -0.05 to -0.01 |
|  | Study day^3^ | 0.002 | 0.002 | 0.001 to 0.003 |
| **Random effects parameter: Pony ID** | Baseline | 6.20 | <0.001 | 6.07 to 6.01 |
|  | Variance (baseline) | 0.03 |  | 0.02 to 0.06 |
|  | Variance (residual) | 0.04 |  | 0.04 to 0.05 |
| **Intraclass correlation** | 0.44 |  |  | 0.28 to 0.61 |

Mixed-effects linear regression models (random effects: Pony ID) were built with volatile fatty acids and pH as the outcome variables and study day as a cubic polynomial as the explanatory variable. An unstructured covariance matrix was employed for the random effects. Coefficients and the intraclass correlation coefficients are presented ± 95% confidence intervals.
