# Supplementary material for: Effect of age and the individual on the gastrointestinal bacteriome of ponies fed a high-starch diet
Source: PLoS One. 2020 May 8;15(5):e0232689. doi: 10.1371/journal.pone.0232689 (PMC7209120; doi:10.1371/journal.pone.0232689)
Supplement: S4 Table — Relative abundance of bacterial phyla and genera across the 11 study days identified to be significantly altered by study day following REML analysis. SED = standard error of the difference. (DOCX) [file pone.0232689.s004.docx]

**Table S4: The relative abundance of bacterial phyla and genera significantly affected by study day.**

|  | **Study day** | | | | | | | | | | | **SED** | **Benjamini-Hochberg P-value** |
| --- | --- | --- | --- | --- | --- | --- | --- | --- | --- | --- | --- | --- | --- |
| **Phylum** | **1** | **2** | **3** | **4** | **5** | **6** | **7** | **8** | **9** | **10** | **11** |  |  |
| *Candidatus Saccharibacteria* | 0.0014 | 0.0015 | 0.0014 | 0.0012 | 0.0014 | 0.0013 | 0.0010 | 0.0010 | 0.0012 | 0.0016 | 0.0015 | 0.0280 | 0.0760 |
| *Fibrobacteres* | 0.1724 | 0.1568 | 0.1760 | 0.1908 | 0.1679 | 0.1542 | 0.1529 | 0.1642 | 0.1737 | 0.1658 | 0.1625 | 0.1920 | 0.0360 |
| *Firmicutes* | 0.2641 | 0.2812 | 0.2709 | 0.2716 | 0.2626 | 0.2794 | 0.2822 | 0.2817 | 0.2703 | 0.2877 | 0.2906 | 0.1100 | 0.0120 |
| **Genus** |  |  |  |  |  |  |  |  |  |  |  |  |  |
| *Phascolarctobacterium* | 0.0128 | 0.0141 | 0.0156 | 0.0129 | 0.0140 | 0.0147 | 0.0156 | 0.0204 | 0.0181 | 0.0144 | 0.0140 | 0.1150 | 0.0173 |
| *Lactobacillus* | 0.0000 | 0.0001 | 0.0001 | 0.0001 | 0.0001 | 0.0003 | 0.0004 | 0.0003 | 0.0005 | 0.0003 | 0.0003 | 0.0220 | 0.0173 |
| *Streptococcus* | 0.0029 | 0.0025 | 0.0017 | 0.0020 | 0.0028 | 0.0226 | 0.0158 | 0.0314 | 0.0279 | 0.0269 | 0.0244 | 0.1900 | 0.0173 |
| *Intestinimonas* | 0.0028 | 0.0025 | 0.0023 | 0.0019 | 0.0026 | 0.0028 | 0.0031 | 0.0043 | 0.0035 | 0.0040 | 0.0030 | 0.0420 | 0.0390 |
| *Fibrobacter* | 0.1779 | 0.1861 | 0.1868 | 0.2071 | 0.1682 | 0.1534 | 0.1660 | 0.1426 | 0.1436 | 0.1477 | 0.1692 | 0.1930 | 0.0594 |
| *Oscillibacter* | 0.0069 | 0.0071 | 0.0071 | 0.0073 | 0.0074 | 0.0076 | 0.0073 | 0.0101 | 0.0078 | 0.0083 | 0.0085 | 0.0370 | 0.0594 |
| *Clostridium IV* | 0.0019 | 0.0025 | 0.0019 | 0.0021 | 0.0023 | 0.0024 | 0.0024 | 0.0028 | 0.0023 | 0.0024 | 0.0022 | 0.0270 | 0.0594 |
| *Sphaerochaeta* | 0.0020 | 0.0027 | 0.0028 | 0.0020 | 0.0021 | 0.0020 | 0.0018 | 0.0018 | 0.0016 | 0.0020 | 0.0016 | 0.0280 | 0.0924 |
| *Anaerocella* | 0.0010 | 0.0007 | 0.0006 | 0.0005 | 0.0007 | 0.0006 | 0.0006 | 0.0006 | 0.0006 | 0.0005 | 0.0005 | 0.0240 | 0.0924 |

Relative abundance of bacterial phyla and genera across the 11 study days identified to be significantly altered by study day following REML analysis. SED = standard error of the difference.
