# Supplementary material for: Effect of age and the individual on the gastrointestinal bacteriome of ponies fed a high-starch diet
Source: PLoS One. 2020 May 8;15(5):e0232689. doi: 10.1371/journal.pone.0232689 (PMC7209120; doi:10.1371/journal.pone.0232689)
Supplement: S5 Table — Mixed-effects linear regression models (random effects: Pony ID) were built with either Streptococcus or Fibrobacter counts (square-root transformed) as the outcome variables and study day as a cubic polynomial as the explanatory variable. An unstructured covariance matrix was employed for the random effects. Coefficients and the intraclass correlation coefficients are presented ± 95% confidence intervals (CI’s). (DOCX) [file pone.0232689.s005.docx]

**Table S5:** **Associations between *Streptococcus, Fibrobacter* and study day**

| **Outcome variable** | **Explanatory variable** | **Coefficient** | **P-value** | **95% confidence interval** |
| --- | --- | --- | --- | --- |
| Square root *Fibrobacter* counts | Study day | 3.65 | 0.02 | 0.49 to 6.81 |
|  | Study day^2^ | -0.87 | 0.005 | -1.47 to -0.27 |
|  | Study day^3^ | 0.05 | 0.003 | 0.02 to 0.08 |
|  | Baseline | 35.96 | <0.001 | 28.90 to 43.01 |
| Random effects parameter: Pony ID | Variance (baseline) | 172.82 |  | 95.78 to 311.80 |
|  | Variance (residual) | 39.69 |  | 33.02 to 47.71 |
| Intraclass correlation | 0.81 |  |  | 0.70 to 0.89 |
| Square root *Streptococcus* counts | Study day | -2.99 | 0.06 | -6.10 to 0.11 |
|  | Study day^2^ | 0.83 | 0.006 | 0.24 to 1.42 |
|  | Study day^3^ | -0.05 | 0.005 | -0.08 to -0.01 |
|  | Baseline | 6.07 | 0.020 | 0.97 to 11.17 |
| Random effects parameter: Pony ID | Variance (baseline) | 34.61 |  | 18.27 to 65.57 |
|  | Variance (residual) | 38.42 |  | 31.96 to 46.18 |
| Intraclass correlation | 0.47 |  |  | 0.32 to 0.64 |

Mixed-effects linear regression models (random effects: Pony ID) were built with either *Streptococcus* or *Fibrobacter* counts (square-root transformed) as the outcome variables and study day as a cubic polynomial as the explanatory variable. An unstructured covariance matrix was employed for the random effects. Coefficients and the intraclass correlation coefficients are presented ± 95% confidence intervals.
