# Supplementary material for: Effect of age and the individual on the gastrointestinal bacteriome of ponies fed a high-starch diet
Source: PLoS One. 2020 May 8;15(5):e0232689. doi: 10.1371/journal.pone.0232689 (PMC7209120; doi:10.1371/journal.pone.0232689)
Supplement: S6 Table — Mixed-effects multivariable regression models were built in with the individual VFA as the outcome variable and the counts of the individual genera (square root transformed) included as explanatory variables and pony identity included as a random intercept. Coefficients ± 95% confidence intervals (95% CI) and p-values (following adjustment for multiple testing using the method proposed by Benjamini and Hochberg to decrease the false discovery rate) are presented. BCVFA: branched chain volatile fatty acids. (DOCX) [file pone.0232689.s006.docx]

**Table S6: Associations between VFA concentrations and the relative abundance of bacterial genera**

|  | **Propionate** | | | **Butyrate** | | | **Acetate** | | | **BCVFA** | | |
| --- | --- | --- | --- | --- | --- | --- | --- | --- | --- | --- | --- | --- |
|  | Coefficient | 95% CI | B-H P value | Coefficient | 95% CI | B-H P value | Coefficient | 95% CI | B-H P value | Coefficient | 95% CI | B-H P value |
| *Acetanaerobacterium* | -0.21 | -0.38 to -0.04 | 0.16 | -0.06 | -0.12  0.01 | 0.41 | -0.42 | -0.91 to  0.07 | 0.42 | -0.34 | -0.51 to  -0.16 | 0.17 |
| *Alkalitalea* | 0.00 | -0.07 to 0.06 | 0.99 | -0.01 | -0.04  0.01 | 0.62 | -0.13 | -0.32 to  0.06 | 0.49 | 0.05 | -0.01 to  0.12 | 0.34 |
| *Alloprevotella* | 0.00 | -0.12 to  0.12 | 0.99 | 0.02 | -0.03 to  0.07 | 0.65 | 0.15 | -0.20 to  0.50 | 0.65 | -0.14 | -0.27 to  -0.01 | 0.25 |
| *Anaerobacterium* | -0.09 | -0.34 to  0.17 | 0.65 | 0.00 | -0.11 to  0.10 | 0.97 | -0.25 | -1.01 to  0.52 | 0.76 | 0.02 | -0.27 to  0.30 | 0.99 |
| *Anaerocella* | 0.00 | -0.17 to  0.18 | 0.99 | 0.01 | -0.06 to  0.08 | 0.93 | 0.09 | -0.42 to  0.59 | 0.87 | -0.01 | -0.19 to  0.17 | 0.99 |
| *Anaeroplasma* | -0.06 | -0.17 to  0.04 | 0.45 | -0.04 | -0.08 to  0.00 | 0.41 | -0.41 | -0.71 to  -0.10 | 0.13 | -0.09 | -0.21 to 0.02 | 0.34 |
| *Anaerorhabdus* | -0.07 | -0.12 to  -0.01 | 0.20 | -0.02 | -0.04 to  0.00 | 0.41 | -0.15 | -0.32 to  0.01 | 0.35 | -0.04 | -0.10 to  0.02 | 0.47 |
| *Anaerotruncus* | -0.02 | -0.25 to  0.21 | 0.96 | 0.04 | -0.04 to  0.12 | 0.62 | 0.27 | -0.35 to  0.89 | 0.65 | -0.15 | -0.36 to  0.05 | 0.36 |
| *Anaerovorax* | 0.09 | -0.20 to  0.38 | 0.68 | 0.05 | -0.07 to  0.18 | 0.63 | 0.26 | -0.64 to  1.16 | 0.77 | 0.44 | 0.09 to  0.79 | 0.17 |
| *Asteroleplasma* | -0.26 | -0.44 to  -0.08 | 0.13 | -0.06 | -0.13 to  0.01 | 0.41 | -0.58 | -1.12 to  -0.05 | 0.17 | -0.25 | -0.46 to  -0.05 | 0.17 |
| *Barnesiella* | -0.02 | -0.08 to  0.03 | 0.65 | 0.00 | -0.02 to  0.02 | 0.97 | 0.06 | -0.09 to  0.22 | 0.66 | -0.01 | -0.07 to  0.04 | 0.91 |
| *Blautia* | 0.02 | -0.23 to  0.26 | 0.98 | -0.03 | -0.13 to  0.07 | 0.78 | -0.09 | -0.84 to  0.66 | 0.92 | -0.27 | -0.55 to  0.02 | 0.29 |
| *Butyricicoccus* | -0.11 | -0.31 to  0.09 | 0.45 | -0.05 | -0.12 to  0.03 | 0.57 | -0.46 | -1.03 to  0.11 | 0.43 | -0.01 | -0.22 to  0.19 | 0.99 |
| *Butyrivibrio* | 0.03 | -0.05 to  0.11 | 0.65 | 0.00 | -0.03 to  0.04 | 0.97 | -0.02 | -0.27 to  0.23 | 0.97 | -0.03 | -0.13 to  0.07 | 0.86 |
| *Candidatusendomicrobium* | -0.11 | -0.28 to  0.06 | 0.45 | -0.05 | -0.12 to  0.02 | 0.47 | -0.36 | -0.88 to  0.16 | 0.49 | -0.08 | -0.27 to  0.12 | 0.78 |
| *Catabacter* | -0.09 | -0.23 to  0.04 | 0.45 | -0.02 | -0.08 to  0.04 | 0.77 | -0.26 | -0.68 to  0.16 | 0.57 | -0.13 | -0.29 to  0.04 | 0.36 |
| *Catenibacterium* | -0.15 | -0.35 to  0.05 | 0.43 | 0.02 | -0.06 to  0.10 | 0.79 | -0.15 | -0.74 to  0.44 | 0.77 | 0.03 | -0.19 to  0.25 | 0.98 |
| *Christensenella* | 0.22 | 0.02 to  0.42 | 0.20 | 0.04 | -0.04 to  0.12 | 0.62 | 0.24 | -0.36 to  0.84 | 0.66 | 0.11 | -0.12 to  0.34 | 0.69 |
| *Clostridium IV* | -0.07 | -0.28 to  0.14 | 0.65 | -0.04 | -0.13 to  0.04 | 0.62 | -0.48 | -1.12 to  0.15 | 0.47 | -0.01 | -0.26 to  0.24 | 0.99 |
| *Clostridium XIVa* | -0.25 | -0.40 to  -0.10 | 0.10 | -0.08 | -0.14 to  -0.02 | 0.29 | -0.78 | -1.23 to  -0.34 | 0.10 | -0.07 | -0.23 to  0.10 | 0.78 |
| *Coprobacter* | 0.06 | -0.03 to  0.15 | 0.45 | 0.00 | -0.03  0.04 to | 0.93 | 0.07 | -0.18 to  0.32 | 0.77 | 0.05 | -0.05 to  0.14 | 0.67 |
| *Ethanoligenens* | -0.08 | -0.22 to  0.06 | 0.45 | -0.04 | -0.09 to  0.01 | 0.41 | -0.19 | -0.59 to  0.21 | 0.62 | 0.14 | 0.00 to  0.28 | 0.28 |
| *Faecalicoccus* | 0.11 | -0.08 to  0.31 | 0.45 | 0.05 | -0.03 to  0.12 | 0.56 | 0.61 | 0.05 to  1.17 | 0.17 | 0.03 | -0.17 to  0.24 | 0.94 |
| *Faecalitalea* | -0.10 | -0.22 to  0.03 | 0.43 | -0.06 | -0.11 to  -0.01 | 0.29 | -0.41 | -0.77 to  -0.05 | 0.17 | -0.01 | -0.14 to  0.11 | 0.99 |
| *Fibrobacter* | -0.04 | -0.08 to  0.00 | 0.32 | -0.01 | -0.03 to  0.01 | 0.60 | -0.06 | -0.18 to  0.06 | 0.62 | -0.04 | -0.08 to  0.01 | 0.34 |
| *Intestinimonas* | 0.10 | -0.01 to  0.21 | 0.32 | 0.02 | -0.03 to  0.06 | 0.74 | 0.19 | -0.14 to  0.52 | 0.57 | 0.15 | 0.03 to  0.28 | 0.17 |
| *Lachnobacterium* | -0.12 | -0.29 to  0.06 | 0.45 | -0.02 | -0.09 to  0.05 | 0.78 | 0.01 | -0.52 to  0.54 | 0.99 | -0.06 | -0.27 to  0.14 | 0.86 |
| *Lachnospiracea_incertae* | 0.06 | -0.12 to  0.23 | 0.65 | 0.02 | -0.05 to  0.09 | 0.79 | 0.30 | -0.23 to  0.84 | 0.57 | 0.19 | -0.02 to  0.39 | 0.29 |
| *Lactobacillus* | -0.26 | -0.47 to  -0.05 | 0.16 | -0.07 | -0.15 to  0.02 | 0.41 | -0.35 | -0.99 to  0.28 | 0.57 | -0.02 | -0.27 to  0.22 | 0.99 |
| *Mobilitalea* | 0.11 | -0.08 to  0.30 | 0.45 | 0.00 | -0.08 to  0.07 | 0.97 | -0.20 | -0.76 to  0.37 | 0.73 | -0.21 | -0.43 to  0.00 | 0.26 |
| *Mogibacterium* | -0.21 | -0.44 to  0.01 | 0.32 | -0.06 | -0.15 to  0.03 | 0.49 | -0.49 | -1.16 to  0.18 | 0.49 | 0.06 | -0.20 to  0.31 | 0.89 |
| *Oscillibacter* | -0.07 | -0.20 to  0.06 | 0.45 | -0.05 | -0.10 to  0.00 | 0.39 | -0.50 | -0.87 to  -0.12 | 0.13 | -0.18 | -0.32 to  -0.05 | 0.17 |
| *Paludibacter* | 0.05 | -0.01 to  0.11 | 0.33 | -0.01 | -0.02 to  0.01 | 0.78 | -0.08 | -0.23 to  0.06 | 0.57 | -0.04 | -0.08 to  0.01 | 0.34 |
| *Papillibacter* | -0.24 | -0.53 to  0.05 | 0.36 | -0.06 | -0.17 to  0.06 | 0.62 | -0.01 | -0.89 to  0.86 | 0.99 | 0.01 | -0.32 to  0.35 | 0.99 |
| *Paraprevotella* | 0.10 | -0.01 to  0.22 | 0.32 | 0.04 | 0.01 to  0.08 | 0.29 | 0.33 | 0.03 to  0.62 | 0.17 | -0.04 | -0.13 to  0.05 | 0.77 |
| *Parvibacter* | -0.28 | -0.48 to  -0.07 | 0.14 | -0.09 | -0.17 to  -0.01 | 0.29 | -0.81 | -1.40 to  -0.21 | 0.13 | -0.18 | -0.40 to  0.04 | 0.34 |
| *Phascolarctobacterium* | 0.05 | -0.05 to  0.15 | 0.52 | 0.02 | -0.02 to  0.06 | 0.63 | -0.03 | -0.33 to  0.27 | 0.92 | -0.03 | -0.14 to  0.09 | 0.89 |
| *Phocaeicola* | -0.05 | -0.13 to  0.02 | 0.45 | -0.03 | -0.06 to  0.00 | 0.41 | -0.15 | -0.36 to  0.07 | 0.49 | -0.04 | -0.12 to  0.04 | 0.66 |
| *Prevotella* | 0.09 | 0.01 to  0.17 | 0.21 | 0.02 | -0.02 to  0.05 | 0.62 | 0.00 | -0.24 to  0.25 | 0.99 | -0.02 | -0.11 to  0.07 | 0.89 |
| *Pseudoflavonifractor* | 0.00 | -0.07 to  0.07 | 0.99 | 0.03 | 0.00 to  0.06 | 0.29 | 0.25 | 0.04 to  0.45 | 0.17 | 0.01 | -0.07 to  0.09 | 0.93 |
| *Pyramidobacter* | -0.14 | -0.48 to  0.19 | 0.60 | 0.03 | -0.10 to  0.17 | 0.79 | 0.56 | -0.44 to  1.56 | 0.57 | 0.31 | -0.06 to  0.69 | 0.34 |
| *Rikenella* | 0.01 | -0.07 to  0.10 | 0.95 | 0.02 | -0.01 to  0.05 | 0.57 | 0.21 | -0.04 to  0.46 | 0.42 | 0.03 | -0.06 to  0.13 | 0.81 |
| *Ruminococcus* | -0.05 | -0.14 to  0.04 | 0.45 | 0.03 | -0.01 to  0.07 | 0.41 | 0.13 | -0.15 to  0.40 | 0.62 | -0.05 | -0.16 to  0.06 | 0.69 |
| *Saccharibacteria_genera* | -0.09 | -0.25 to  0.06 | 0.45 | -0.05 | -0.11 to  0.02 | 0.46 | -0.13 | -0.60 to  0.33 | 0.77 | -0.18 | -0.35 to  -0.01 | 0.25 |
| *Sphaerochaeta* | -0.04 | -0.18 to  0.10 | 0.71 | 0.02 | -0.03 to  0.07 | 0.65 | 0.09 | -0.30 to  0.49 | 0.77 | -0.04 | -0.17 to  0.10 | 0.89 |
| *Sporobacter* | 0.07 | -0.14 to  0.27 | 0.65 | 0.00 | -0.08 to  0.09 | 0.97 | 0.29 | -0.32 to  0.90 | 0.62 | 0.05 | -0.19 to  0.29 | 0.89 |
| *SR1_genera_incertae_sedis* | 0.16 | 0.02 to  0.30 | 0.20 | 0.05 | 0.00 to  0.11 | 0.41 | 0.48 | 0.04 to  0.91 | 0.17 | 0.17 | 0.01 to  0.34 | 0.25 |
| *Streptococcus* | 0.00 | -0.04 to  0.03 | 0.95 | 0.00 | -0.01 to  0.01 | 0.99 | 0.01 | -0.09 to  0.11 | 0.92 | -0.02 | -0.06 to  0.01 | 0.51 |
| *Treponema* | -0.04 | -0.11 to  0.04 | 0.54 | -0.02 | -0.05 to  0.01 | 0.41 | 0.00 | -0.21 to  0.20 | 0.99 | 0.02 | -0.06 to  0.09 | 0.89 |
| *Unclassified* | -0.01 | -0.08 to  0.07 | 0.98 | 0.00 | -0.03 to  0.03 | 0.98 | 0.06 | -0.17 to  0.29 | 0.77 | 0.00 | -0.09 to  0.08 | 0.99 |
| *Ureaplasma* | -0.06 | -0.21 to  0.08 | 0.60 | -0.01 | -0.07 to  0.04 | 0.79 | -0.10 | -0.50 to  0.30 | 0.77 | -0.15 | -0.29 to  -0.01 | 0.25 |
| *Vampirovibrio* | -0.10 | -0.25 to  0.05 | 0.45 | -0.03 | -0.09 to  0.03 | 0.62 | -0.23 | -0.70 to  0.23 | 0.62 | 0.00 | -0.18 to  0.18 | 0.99 |
| Baseline | 11.54 | 3.15 to  19.92 |  | 3.27 | -0.04 to  6.57 |  | 23.59 | -1.01 to  48.19 |  | 7.12 | -1.80 to  16.04 |  |

Mixed-effects multivariable regression models were built in with the individual VFA as the outcome variable and the counts of the individual genera (square root transformed) included as explanatory variables and pony identity included as a random intercept. Coefficients ± 95% confidence intervals (95% CI) and p-values (following adjustment for multiple testing using the method proposed by Benjamini and Hochberg to decrease the false discovery rate) are presented. BCVFA: branched chain volatile fatty acids.
