# Supplementary material for: Effect of age and the individual on the gastrointestinal bacteriome of ponies fed a high-starch diet
Source: PLoS One. 2020 May 8;15(5):e0232689. doi: 10.1371/journal.pone.0232689 (PMC7209120; doi:10.1371/journal.pone.0232689)
Supplement: S7 Table — Separate logistic regression models were built with the binary variable ‘responder’ (based on raw Streptococcus counts) as the outcome variable and individual phenotype parameters (including CGIT parameters, outset bacterial diversity and outset faecal VFA/pH) as the explanatory variables. (DOCX) [file pone.0232689.s007.docx]

**Table S7: Outset phenotype parameters in ‘responders’ or ‘non-responders’**

| **Explanatory variable** | **Odds ratio** | **P value** | **95% confidence interval** |
| --- | --- | --- | --- |
| Outset %body fat  Baseline | 1.42  0.0003 | 0.11  0.08 | 0.92 to 2.21  0.003 to 2.55 |
| Baseline insulin  Baseline | 1.15  0.06 | 0.09  0.003 | 0.98 to 1.34  0.008 to 0.39 |
| Insulin time 45  Baseline | 1.01  0.04 | 0.10  0.008 | 1.00 to 1.03  0.003 to 0.42 |
| Insulin time 75  Baseline | 1.01  0.09 | 0.27  0.004 | 0.99 to 1.03  0.02 to 0.47 |
| AUC insulin  Baseline | 1.00  0.04 | 0.11  0.007 | 0.99 to 1.00  0.004 to 0.42 |
| Baseline glucose  Baseline | 1.27  0.04 | 0.83  0.59 | 0.15 to 10.81  0.004 to 4852.19 |
| AUC glucose  Baseline | 1.00  0.11 | 0.93  0.51 | 0.99 to 1.01  0.002 to 74.36 |
| Return to baseline glucose concentration  Baseline | 1.00  0.11 | 0.78  0.07 | 0.98 to 1.03  0.01 to 1.18 |
| Outset pH  Baseline | 9.59  6.98e-08 | 0.39  0.33 | 0.06 to 1578.63  2.59e-22 to 1.83e+07 |
| Outset Simpson  Baseline | 2.98e+09  8.14e-11 | 0.52  0.48 | 9.23e-20 to 9.63e+37  6.34e-39 to 1.04e+18 |
| Outset Shannon  Baseline | 2.01  0.002 | 0.50  0.33 | 0.27 to 15.08  1.34e-08 to 461.46 |
| Outset Chao1  Baseline | 1.00  0.12 | 0.93  0.42 | 0.99 to 1.00  0.001 to 20.30 |
| Outset Sobs  Baseline | 1.00  0.01 | 0.47  0.22 | 1.00 to 1.01  0.001 to 12.41 |
| Outset acetate  Baseline | 1.06  0.08 | 0.71  0.19 | 0.78 to 1.43  0.002 to 3.41 |
| Outset butyrate  Baseline | 1.40  0.10 | 0.75  0.14 | 0.17 to 11.28  0.004 to 2.13 |
| Outset propionate  Baseline | 1.71  0.02 | 0.11  0.02 | 0.89 to 3.27  0.001 to 0.49 |

Separate logistic regression models were built with the binary variable ‘responder’ (based on raw *Streptococcus* counts) as the outcome variable and individual phenotype parameters (including CGIT parameters, outset bacterial diversity and outset faecal VFA/pH) as the explanatory variables.
