# Supplementary material for: Effect of age and the individual on the gastrointestinal bacteriome of ponies fed a high-starch diet
Source: PLoS One. 2020 May 8;15(5):e0232689. doi: 10.1371/journal.pone.0232689 (PMC7209120; doi:10.1371/journal.pone.0232689)
Supplement: S8 Table — (DOCX) [file pone.0232689.s008.docx]

**Table S8: Core OTU population**

|  | **OTU** | **Phylum** | **Class** | **Order** | **Family** | **Genus** |
| --- | --- | --- | --- | --- | --- | --- |
| **All samples** | OTU_35 | Bacteroidetes | Bacteroidia | Bacteroidales | Prevotellaceae | Prevotella |
| **Hay only samples** | OTU_16 | Bacteroidetes | Bacteroidia | Bacteroidales | Porphyromonadaceae | unclassified |
|  | OTU_40 | Unclassified | Unclassified | Unclassified | Unclassified | Unclassified |
|  | OTU_90 | Bacteroidetes | Bacteroidia | Bacteroidales | Unclassified | Unclassified |
|  | OTU_44 | Firmicutes | Negativicutes | Selenomonadales | Unclassified | Unclassified |
|  | OTU_99 | Proteobacteria | Unclassified | Unclassified | Unclassified | Unclassified |
|  | OTU_13 | Bacteroidetes | Bacteroidia | Unclassified | Unclassified | Unclassified |
|  | OTU_51 | Spirochaetes | Spirochaetia | Spirochaetales | Spirochaetaceae | Treponema |
|  | OTU_130 | Firmicutes | Clostridia | Clostridiales | Ruminococcaceae | Intestinimonas |
|  | OTU_35 | Bacteroidetes | Bacteroidia | Bacteroidales | Prevotellaceae | Prevotella |
| **Barley only samples** | OTU_35 | Bacteroidetes | Bacteroidia | Bacteroidales | Prevotellaceae | Prevotella |
