# Supplementary material for: Effect of age and the individual on the gastrointestinal bacteriome of ponies fed a high-starch diet
Source: PLoS One. 2020 May 8;15(5):e0232689. doi: 10.1371/journal.pone.0232689 (PMC7209120; doi:10.1371/journal.pone.0232689)
Supplement: S9 Table — Summary of ANOSIM and PERMANOVA outputs. Significant P-values for PERMANOVA (p < 0.05) are highlighted. ANOSIM R-values indicate the degree of separation between samples (0 = very similar; 1 = highly dissimilar), with significant R-values (with p < 0.05) shown in bold. (DOCX) [file pone.0232689.s009.docx]

**Table S9. Effect of group (Control and Aged) and diet (hay and hay + barleyon the structure of the bacterial communities in the faeces.**

|  | PERMANOVA (P-value) | ANOSIM (R-value) |
| --- | --- | --- |
|  |  |  |
| Group (Control and Aged) | 0.001 | **0.151** |
| Diet | 0.404 | 0 |
| Group x diet | 0.943 | 0.100 |

Significant P-values for PERMANOVA (p < 0.05) are highlighted. ANOSIM

R-values indicate the degree of separation between samples (0 = very similar;

1 = highly dissimilar), with significant R-values (with p < 0.05) shown in bold.
