# Supplementary figures and images for: Effect of age and the individual on the gastrointestinal bacteriome of ponies fed a high-starch diet
Source: PLoS One. 2020 May 8;15(5):e0232689. doi: 10.1371/journal.pone.0232689 (PMC7209120; doi:10.1371/journal.pone.0232689)

**Figure S1: Rarefaction curves**

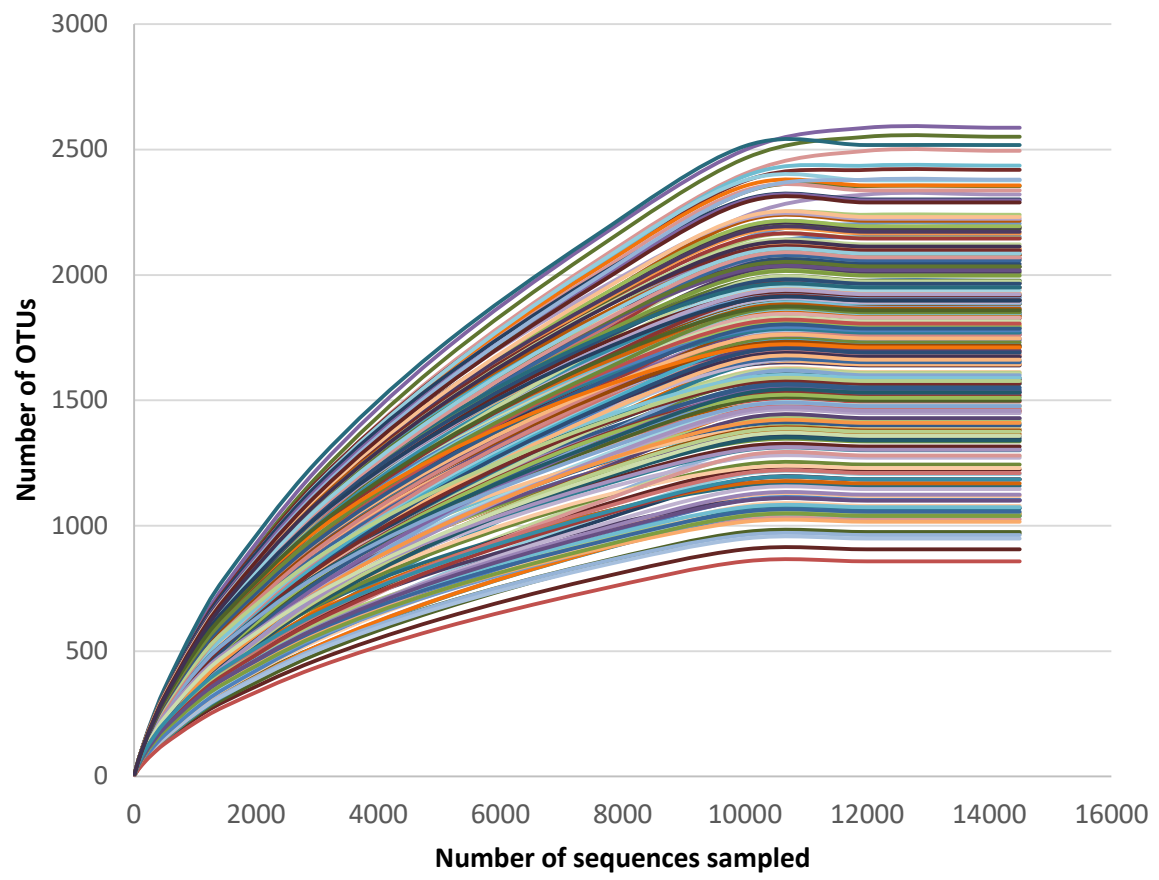

Supplement: S1 Fig — (PDF) [file pone.0232689.s010.pdf]

**Figure S3: Jaccard index between the individual study days for the Control and Aged groups**

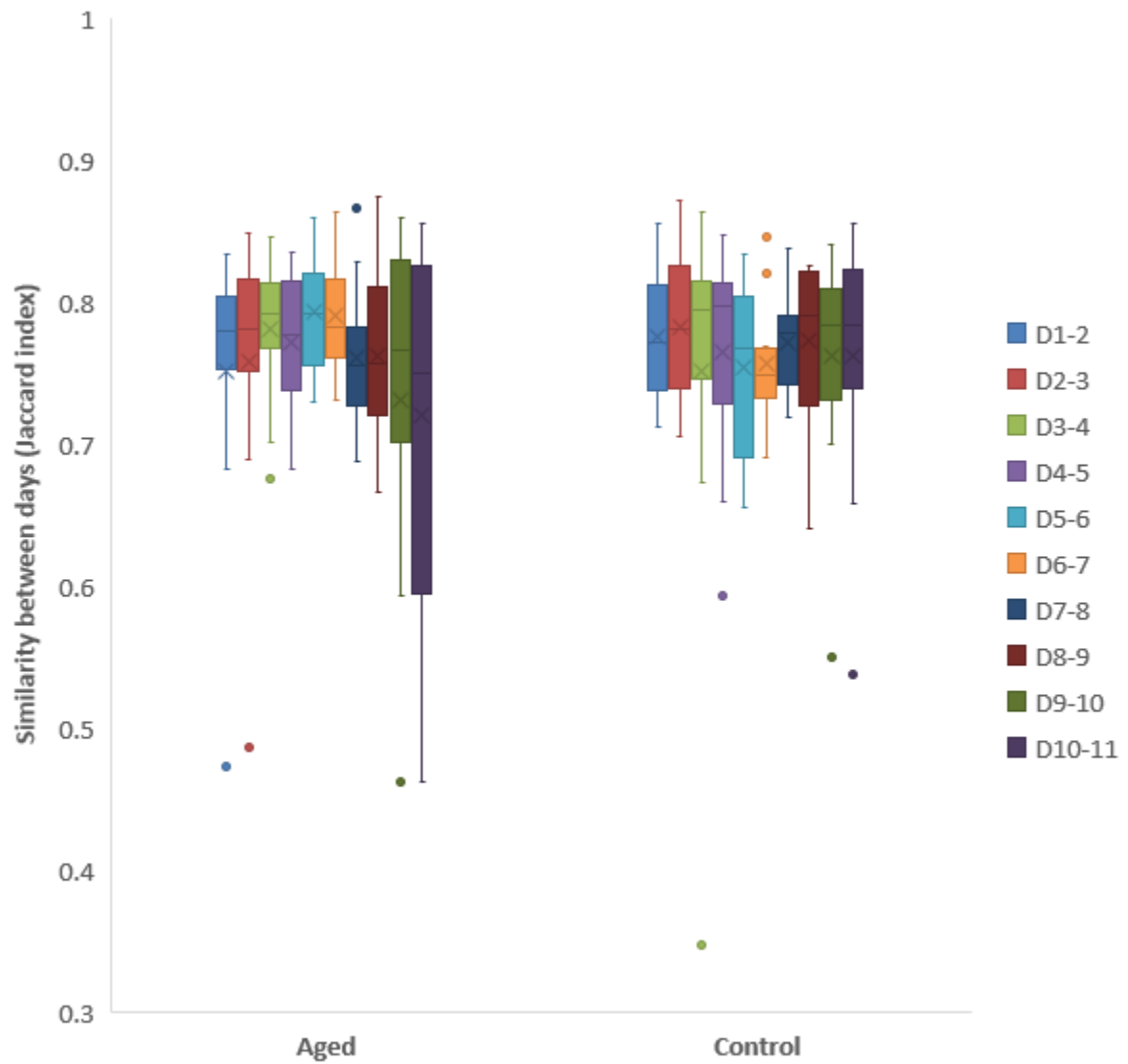

Supplement: S3 Fig — (PDF) [file pone.0232689.s012.pdf]
