# Supplementary material for: Effect of age and the individual on the gastrointestinal bacteriome of ponies fed a high-starch diet
Source: PLoS One. 2020 May 8;15(5):e0232689. doi: 10.1371/journal.pone.0232689 (PMC7209120; doi:10.1371/journal.pone.0232689)

Figure S2: Non-metric multi-dimensional scaling plot of the bacterial OTU data (excluding those OTU's present at <0.01% relative abundance) by group and diet

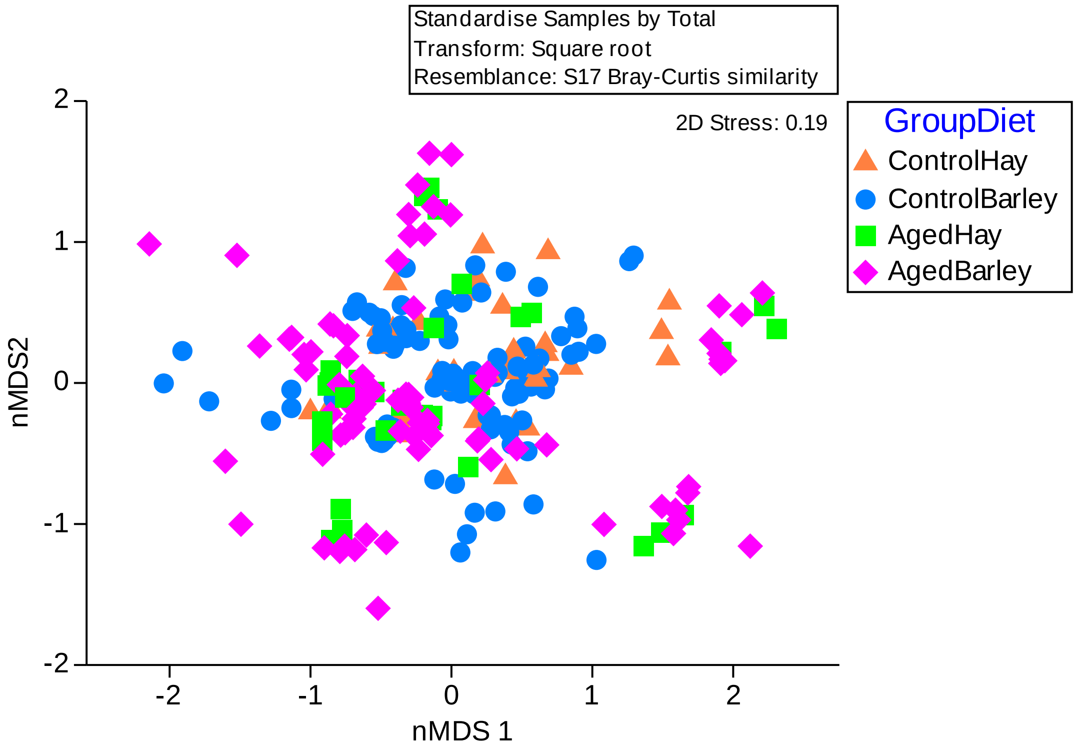

Supplement: S2 Fig — (PDF) [file pone.0232689.s011.pdf]
